# World Trade Center Dust Exposure Promotes Cancer in PTEN-deficient Mouse Prostates

**DOI:** 10.1158/2767-9764.CRC-21-0111

**Published:** 2022-06-27

**Authors:** Lin Wang, Yitian Xu, Licheng Zhang, Kyeongah Kang, Andriy Kobryn, Kensey Portman, Ronald E Gordon, Ping-Ying Pan, Emanuela Taioli, Stuart A Aaronson, Shu-Hsia Chen, David J Mulholland

**Affiliations:** 1 Department of Oncological Sciences, Icahn School of Medicine at Mount Sinai, New York, New York.; 2 Center for Immunotherapy Research, Cancer Center of Excellence, Houston Methodist Research Institute, Houston, Texas.; 3 Department of Pathology, Icahn School of Medicine, New York, New York.; 4 Institute for Translational Epidemiology, Icahn School of Medicine at Mount Sinai, New York, New York.; 5 Tisch Cancer Institute, New York, New York.

## Abstract

During the 9/11 attacks, individuals were exposed to World Trade Center (WTC) dust which contained a complex mixture of carcinogens. Epidemiologic studies have revealed the increased incidence of prostate and thyroid cancer in WTC survivors and responders. While reports have shown that WTC-dust associates with the increased prevalence of inflammatory-related disorders, studies to date have not determined whether this exposure impacts cancer progression. In this study, we have used genetically engineered mouse (GEM) models with prostate-specific deletion of the PTEN tumor suppressor to study the impact of WTC-dust exposure on deposition of dust particles, inflammation, and cancer progression. In normal C57/BL6 mice, dust exposure increased cellular expression of inflammatory genes with highest levels in the lung and peripheral blood. In normal and tumor-bearing GEM mice, increased immune cell infiltration to the lungs was observed. Pathologic evaluation of mice at different timepoints showed that WTC-dust exposure promoted PI3K-AKT activation, increased epithelial proliferation and acinar invasion in prostates with heterozygous and homozygous *Pten* loss. Using autochthonous and transplant GEM models of prostate cancer, we demonstrated that dust exposure caused reduced survival as compared with control cohorts. Finally, we used imaging mass cytometry to detect elevated immune cell infiltration and cellular expression of inflammatory markers in prostate tumors isolated from human WTC survivors. Collectively, our study shows that chronic inflammation, induced by WTC dust exposure, promotes more aggressive cancer in genetically predisposed prostates and potentially in patients.

**Significance:**

We provide the first evidence that exposure to WTC dust promotes prostate cancer progression. These data may impact the diagnoses, clinical management, and treatment of responders who have or will develop cancer.

## Introduction

World Trade Center (WTC) dust is comprised of a complex mixture of asbestos, silica, benzene, polychlorinated biphenyls, polycyclic aromatic hydrocarbons, volatile organic compounds, and metals (1–3). The presence of such carcinogens has raised the possibility that individuals exposed to the WTC environment after its collapse could have an increased risk of cancer. To date, epidemiologic studies have shown increased cancer rates of 6%–19% above background with the highest increases for prostate and thyroid cancer (4–7). These increases have been confirmed in subsequent studies involving individuals outside of the rescue recovery and hold true even after considering factors such as surveillance bias (8). However, the complexity in understanding the association between acute dust exposure and cancer is confounded by the latency between exposure time and development of disease. Consequently, there is a critical need for *in vivo* functional experiments, to dissect the effects of dust exposure on cancer development in a more rapid and controlled manner. Previous studies have shown that acute WTC dust exposure in the rat alters expression of genes involved in oxidative stress and inflammatory/immune functions (9, 10). Nonetheless, while it is understood that inflammation can contribute to tumorigenesis (11–13), studies to date have not experimentally linked WTC dust exposure with cancer promotion. In this study, we use a preclinical mouse model of prostate cancer, with loss of the PTEN tumor suppressor (14), to address whether WTC dust exposure can impact the organ distribution of cancer-causing dust components, the association with inflammation and disease progression.

## Materials and Methods

### Ethics

All mouse modeling studies conducted in this study were performed according to an approved Institutional Animal Care and Use Committee (IACUC) protocol (LA13-00060).

### Inclusion and Exclusion Criteria

Female mice were excluded from animal studies as this study focuses on prostate cancer which is a male only disease.

### Attrition

This is a preclinical study and therefore the attrition rate is defined only when mice are found dead or moribund.

### Sex as a Biological Variable

This study uses male and female mice for breeding of experimental C57/BL6 mice. However, all experimental mice are males as prostate cancer is a male only disease.

### Randomization

Autochthonous and transplant model of prostate cancer were randomly assigned for WTC dust or PBS control treatment using the RAND function on excel.

### Blinding

Tumor measurements are completed by a dedicated technician who is blind to the treatment IDs of tumor-bearing mice.

### Replication

All *in vivo* dosing studies were completed with five replicates per treatment cohort, and three times for replication of the study (defined by different animal cohorts). Flow cytometry analysis is conducted with three study replicates. Aggregate data for flow cytometry, IHC analysis, and q-PCR are also a result of at least three independent experimental replicates or mice analyzed.

### Animal Studies

All animal experiments were approved under the IACUC protocol LA13-00060.

### GEM Mice

PB-Cre4 mice (01XF5) and *Pten*-loxp (exon4) mice were obtained from the NCI Frederick repository. To generate mice with prostate specific deletion of *Pten*, PB-Cre mice were crossed with *Pten*-loxp mice to generate mice with heterozygous (*Pb-Cre^+^;Pten^L^^/Wt^*) and homozygous deletion (*Pb-Cre^+^;Pten^L/L^*).

### Transplant Models

Primary GEM tumors were resected and dissociated to the single-cell level using mechanical and enzymatic dissociation. PureCol-coated tissue culture plates (0.1 mg/mL) were washed 3× with dH_2_O and plated with single-cell dissociates of tumor cells from Pten-deficient mouse prostates. After expansion through 2–3 cell passages, cells were collected and used for implantations to C57BL/6 mice. For intraprostate implants, cell-Matrigel (BD Biosciences) composites were generated at a concentration of 10–50 K per 5 μL injected volume. In brief, animals were sedated using ketamine (50 mg/mL) and xylazine (20 mg/mL), shaved and prostate accessed by midline incision. Using Hamilton microsyringes, each injected prostate received 2 × 5 μL cell-Matrigel injections including one injection in each prostate lobe. For venous injections, cells were made up in media at a concentration of 50–200 K per 100 μL injectable volume and injected to the lateral vein of immobilized mice under a heat lamp.

### Human Study Participants

Responders who participated (as employees or volunteers) in the rescue, recovery, and cleanup efforts at the WTC sites were enrolled at Mount Sinai in the World Trade Center Health Program (WTCHP), which is funded under the James Zadroga 9/11 Health and Compensation Act of 2010, on the basis of eligibility criteria including type of duties, site location, and dates and hours worked (15). The medical protocol for the monitoring program includes self-administered physical and mental health questionnaires as well as a physical examination, laboratory tests, spirometry, and a chest radiograph (22). Of the 27,000 responders that have had a least one monitoring visit in the WTCHP, 17,781 are male responders who have consented to have their records used for medical research (16).

### Human Sample Acquisition

Patient recruitment and sample retrieval has been described previously (15). In brief, patients were contacted by letter and/or by phone to give written study consent, and formalin-fixed paraffin-embedded tumor-tissue samples were requested from the hospitals where they received their prostate cancer treatment. Non-WTC prostate cancer samples were obtained from the Mount Sinai tumor tissue bank. Of the originally included 17 WTC patients and 17 non-WTC patients, frequency matched on age (± 5 years), race/ethnicity and Gleason score. The use of all human samples was approved by Institutional Review Board.

### Tumor Cell Isolation

Human and mouse tumors were then resected and dissociated to the single-cell level in a solution of RPMI—10% FBS (Gibco) + 1% type 1 collagenase (Gibco). The dissociate is subject to mechanical dissociation through a 16G needle and syringe (5–10 passes) and then a 21.5G needle (5–10 passes). Single-cell dissociates were then sorted for live CD45-positive cells using a BD Aria after cell staining using a BV510-CD45 antibody (BioLegend) and DAPI stain. Single-cell suspensions of all sorted samples were then resuspended in BSA (0.2% in PBS) at a concentration of 2 × 10^6^ cells/mL followed by barcoding with a 10× Chromium Controller (10× Genomics).

### WTC Dust Dosing

The administration of WTC dust was conducted according to previously established studies (17, 18). In brief, we obtained WTC dust from Clifford Weisel (Rutgers University, New Brunswick, NJ) which was sonicated for up to 30 seconds and reconstituted in PBS at concentrations of up to 5 mg/25 μL inoculums. To introduce the WTC dust–PBS mixture to C57BL/6 mice, the inoculum was applied to the naris of each mouse. When the inoculum disappeared, the mouse was considered to have been successfully dosed. The size of WTC dust particles has been previously reported to be 80%–90% >2 μm (19) and 50% >53 μm (1, 20).

### Gene Expression

Gene expression analysis was conducted using the 96-well RT2 profiler PCR Array which contains a set of 84 related genes, five housekeeping genes, and three controls (Qiagen). In brief, RNA-cDNA was produced for control- and WTC-treated tissue mouse tissues using standard procedures. cDNA was then added to the RT2 SYBR Green Master Mix and aliquoted across the RT2 Profiler Array and run in by real-time PCR. Software analysis and volcano plots were produced using the RT2 Profiler PCR Array Data Analysis Web portal.

### Flow Cytometry

Flow cytometry analysis was conducted on BD LSR Fortessa (X-20 Cell Analyzer) using BD FACS Diva software (8.0.1).

### Antibodies

The following antibodies were used for flow cytometry: CD45 (BioLegend, 103138), CD3 (BioLegend, 101822), NK1.1 (BioLegend, 156504), CD19 (BioLegend, 118216), P-AKT-S473 (Cell Signaling, #4060), SMA (R&D, MAB1420).

### Pathology and Cell Counting

All tissues and sectioning were processed using the Oncological Sciences Histology Core (Mount Sinai). For cell counting, stained tissue sections were scanned to digital format (Dept of Pathology, Icahn School of Medicine at Mount Sinai, New York, NY) and accessed using QuPath imaging software (21). Values for absolute and percentage cell types (% Ki67 positive) was done using hematoxylin to demark total cells and diaminobenenzidine (DAB) positive cells to demark antibody reactive cells.

### Mass Spectrometry

Heavy metal analysis was conducted by Lung-Chi Chen at NYU Langone (10).

### Asbestos Fiber Microscopy

Fiber particle analysis was conducted by Dr. Ronald Gordon, (Department of Pathology, Icahn School of Medicine at Mount Sinai, New York, NY) and was conducted using methods as described previously (42). In brief, WTC and control samples were received fixed in formalin. Portions of lung tissue were removed. The tissue from each tissue type was weighed after blotting away the excess fluid. All the tissue types were kept separate throughout the study. The tissues were minced and then submerged in 5% KOH for digestion of the biologic material. The digested tissue was centrifuged to separate the nonsolubilized materials from reagents and solubilized materials. The precipitate was washed five times with distilled water. The digested tissue material was resuspended in a known amount of distilled and 10 μL samples were removed from each tissue preparation and placed on formvar-coated nickel grids. The grids were analyzed by transmission electron microscopy. The tissues were counted using a standard fiber counting protocol which including looking at 800 grid openings (42). Asbestos was assessed to determine whether they met the definition of a fiber including having at least a 5:1 length:width ratio and parallel sides of at least 5 μm in length. The fibers were also analyzed by energy dispersive spectroscopy to determine the ratio of elements contained in the fibers and by selected area electron diffraction to confirm the crystalline structure of putative asbestos fibers. Positive controls from the WTC dust and negative control samples prepared from the same distilled water that were used to wash the samples. Verification techniques of fiber counting were used for quality control and quality assurance. The type of asbestos found was determined by comparing with UICC control samples of asbestos.

### Imaging Mass Cytometry and Human Prostate Tissues

Methods were described previously (22). Briefly, metal-labeled antibodies were conjugated according to the Fluidigm protocol. Metal-tagged antibodies used in the imaging mass cytometry (IMC) staining were previously validated with human spleen or thymus tissue in Houston Methodist Immuno Monitoring core before performing the assays. All IMC assays were completed using the same batch of antibodies, titrations, and standard operation protocol–defined conditions. Conjugated metals and antibodies used in this study are listed in Table 1.

**TABLE 1:** Left, List of metal conjugated antibodies used for IMC. Right, List of *t*-SNE clusters and associated cell types.

Metallic agent	Antibody	Cluster	Cell type
Pr141	GramZB	1	Ki67^+^
Nd142	CD20	2	p504S+PanCK+
Nd143	CD3	3	PanCK+
Nd144	CD206	4	p63+
Nd145	CD4	5	Ecad+
Nd146	CD8a	6	CD31^+^
Sm147	SMA	7	SMA+
Nd148	PanCK	8	Collagen+
Sm149	CD31	9	SMA+Collagen+
Nd150	PD-L1	10	CD163^+^CD206^+^
Eu151	PD-1	11	CD86^+^
Sm152	CD163	12	CD68^+^
Eu153	p504S	13	CD4^+^CD3^+^CD45^+^
Sm154	Foxp3	14	CD8^+^CD45^+^CD3^+^
Gd155	pSTAT1	15	CD20^+^CD45^+^
Gd156	CD45	16	CD15^+^
Gd158	E-caderin	17	CD56^+^
Tb159	CD68	18	CD45^+^
Gd160	CD14	19	CD3lo
Dy161	NOS2	20	pp38+
Dy162	NFATC2	21	HLADR+
Dy163	pSTAT3	22	Foxp3+
Dy164	CD15	23	PD1+
Ho165	pERK	24	pSTAT1+
Er166	pNFκB	25	pERK+
Er167	Ki67	26	pAKT+
Er168	pAKT	27	pSTAT3+
Tm169	Collagen	28	pNFkB+
Er170	CD1c	29	Surface neg
Yb171	p63		
Yb172	p-p38		
Yb173	Tbet		
Yb174	HLA-DR		
Lu175	CD86		
Yb176	CD56		

Prostate tissues from 8 WTC patients with prostate cancer and 12 non-WTC prostate cancer cases were obtained from the WTC tissue biobank (23). Three unstained slides from each patient were delivered to Houston Methodist Immuno Monitoring core to perform IMC. One slide of each patient was stained with hematoxylin and eosin (H&E). For staining, tumor sections were baked at 60°C overnight, then dewaxed in xylene and rehydrated in a graded series of alcohol. Heat-induced epitope retrieval was conducted in a temperature-controlled microwave at 95°C in Tris-Tween20 buffer at pH 9 for 20 minutes. After immediate cooling for 20 minutes, the sections were blocked with 3% BSA in TBS for 1 hour. Sections were incubated with an antibody master mix overnight at 4°C. Samples were then washed four times with TBS/0.1% Tween20 and stained with Cell-ID Intercalator (Fluidigm) for 5 minutes. Slides were washed twice with TBS/0.1% Tween20, air dried and stored at 4°C for ablation.

Sections were ablated with Hyperion (Fluidigm) for data acquisition. A total of 1 mm^2^ region of each patient slide was ablated at the border of tumor area in the junction between the tumor and normal stromal cell area to obtain IMC data. IMC data were segmented by Ilastik and CellProfiler. Histology topography cytometry analysis toolbox (histoCAT) and R scripts were used to quantify cell number, generate *t*-SNE plots, and perform neighborhood analysis (24). Slides were subsequently stained with H&E and sent for whole slide scanning at 40× magnification with an Aperio Scanscope 2. The images were viewed in ImageScope.

### Data Processing

Data were converted to TIFF format and segmented into single cells using the flexible analysis pipeline available at https://github.com/BodenmillerGroup/ImcSegmentationPipeline. In brief, individual cells were segmented using a combination of Ilastik v.1.3.3 (3) and CellProfiler v.3.1.8 (25). Ilastik was used to generate a probability map by classifying pixels (single cells: nuclei, membrane, and background) based on a combination of antibody stains to identify membranes and nuclei. Probability maps were then segmented into single-cell object masks using CellProfiler. Single-cell segmentation masks and TIFF images of the 35 channels were overlaid and the mean expression levels of markers and spatial features of single cells were extracted using the MATLAB toolbox region props. Even with very-good-quality segmentation, the imaging of tissue segments results in single-cell data of tissue slices and overlapping cell fragments that do not always capture the nucleus of a cell, and therefore nuclei-mismatched signals can be assigned to neighboring cells in densely packed areas. This can lead to rare cases in which data assigned to one cell contains marker expression from the neighborhood.

### Data Transformation and Normalization

The presented data were not transformed, and all analyses were based on raw IMC measurements. Single-cell marker expressions are summarized by mean pixel values for each channel. The single-cell data were censored at the 99th percentile to remove outliers. For *t*-SNE and PhenoGraph, the data were normalized to the 99th percentile, as is suggested for these algorithms (26, 27). To visualize the number of cells per image or patient, the cell counts were normalized by the image area (total number of pixels, 1 pixel = 1 μm^2^) and displayed as cell density.

### Analysis Workflow

The single-cell analysis pipeline was implemented in R, but image analysis steps were performed in MATLAB. All statistical tests were performed using common functions in R.

### PhenoGraph

PhenoGraph version 0.2 was used (26). Data were 99th percentile normalized before the analysis, and default parameters with nearest neighbors of 35 were used. This parameter was chosen based on prior knowledge of the underlying cell types. The seed used was 2008.

### Barnes–Hut *t*-SNE

For visualization, high-dimensional single-cell data were reduced to two dimensions using the nonlinear dimensionality reduction algorithm *t*-SNE (6). We applied the Barnes–Hut implementation of *t*-SNE to 99th-percentile normalized data with default parameters (initial dimensions, 110; perplexity, 30; *θ*, 0.5). The seed used was 2008.

### Neighborhood Analysis

To identify significantly enriched or depleted pairwise neighbor interactions between cell types, histoCAT functions were used to perform a permutation test–based analysis of spatial single-cell neighborhoods (24). Neighboring cells were defined as those within 4 pixels (4 μm). *P* values smaller than 0.05 were considered as significant.

### Data Availability Statement

Data acquired in this study can be accessed by contacting the corresponding author of this study.

## Results

### Modeling WTC Dust Exposure in the Prostatic Epithelium

**FIGURE 1:**
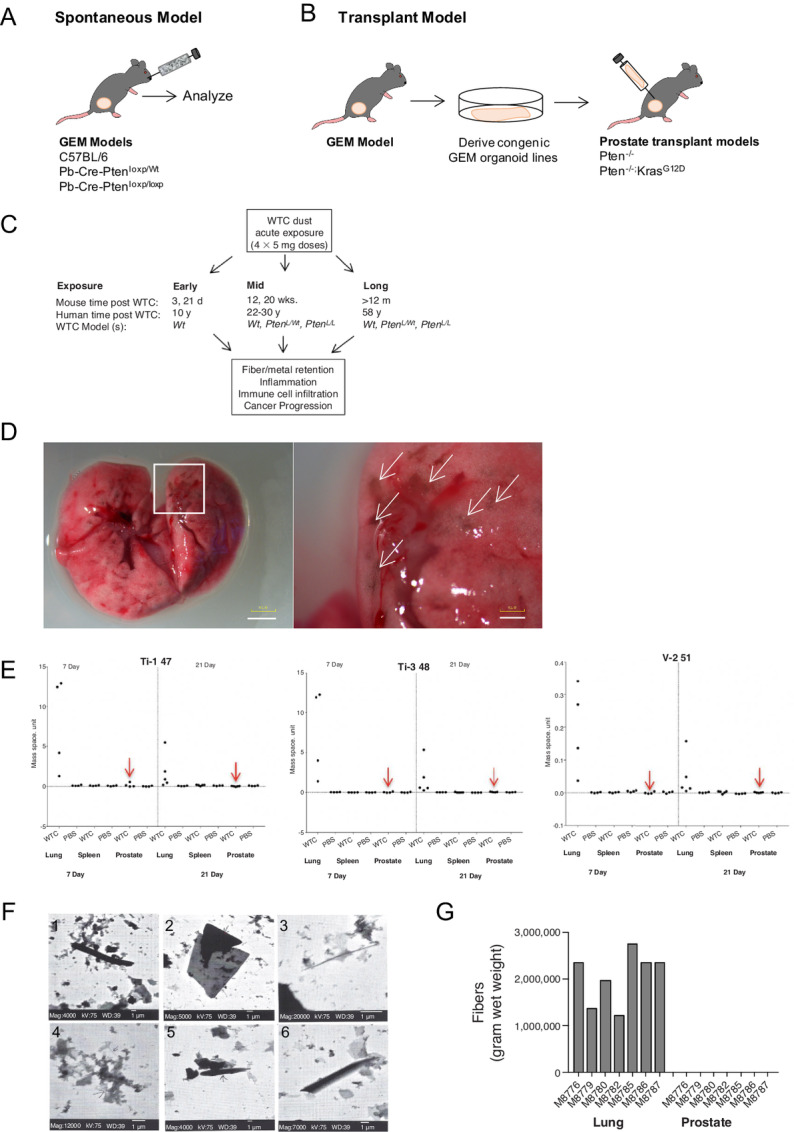
Modeling WTC dust exposure in GEM models of prostate cancer. **A,** Genetic mouse models of autochthonous prostate cancer including those having *Pten Wt* levels (Cre-neg or *Pb-Cre ^+^;Pten^Wt^^/Wt^)* and *Pten loss* (*Pb-Cre^+^;Pten^L/Wt^*, *Pb-Cre^+^;Pten^L/L^*). Early in progression, GEM mice were exposed to WTC dust (4 × 5 mg doses *via* nasal instillation) and evaluated for inflammatory, pathologic and progression changes. **B,** Transplant models of prostate cancer progression using congenic organoid lines derived from donor primary GEM tumor models. Tumor lines were expanded *in vitro* and surgically implanted into the prostates of C57/BL6 mice. Implanted mice were subsequently exposed to WTC dust (4 × 5 mg doses) and evaluated for progression changes. **C,** Cohorts, mice used and measurable outputs at short (3, 21 days), medium (12, 20 weeks) and long (>12 m) timepoints after WTC dust exposure. Human years for each timepoint are shown along with measurable outputs for the analysis. **D,** Representative images of WTC dust–exposed mice showing gross enlargement and retention of WTC dust components (arrows) (bar = 1 mm, left) (bar = 100 μm). **E,** Mass spectrometry analysis showing the retention and quantification of heavy metal components (Ti-147, Ti-3, V-251 in mass spec units) in lung, spleen, and prostate 21 days after WTC dust exposure in C57BL/6 mice (*n* = 12). The black dots represent individual tissues analyzed. **F,** Analytic microscopy, and electron micrographs showing the presence of fibrous WTC dust components 21 days after WTC dust exposure. **G,** Comparison of counted fibers using analytic microscopy in lung and prostate implanted mice (*n* = 8) 21 days after exposure to WTC dust. Fibers were quantitated in the lung and prostate per gram of wet weight tissue (bar = 1 μm).

To model the impact of WTC dust exposure on first responders, we developed mouse models of autochthonous prostate cancer that are deficient for PTEN—a tumor suppressor lost in the majority of human primary and metastatic tumors (28, 29). Using cre-loxp technology, we generated mice with prostate-specific homozygous (*Pb-Cre^+^;Pten^L^^/L^* GEM*)* or heterozygous *Pten* deletion (*Pb-Cre^+^;Pten^L/Wt^* GEM). Changes in the prostatic epithelia of these mice includes progression to high-grade prostatic intraepithelial neoplasia (PIN) or invasive carcinoma by 8–10 weeks or >3 months, respectively (ref. 14; Fig. 1A). For controls, we generated age-matched cre-negative mice which did not have *Pten* loss. Using these GEM primary tumors, we derived C57BL/6 prostate organoids which were expanded *in vitro* and subsequently transplanted to male C57BL/6 mice by three injection routes including orthotopic implantation to the proximal prostate (10–50 K cells/5 μL implant; ref. 30), venous injection (lateral tail vein, 50–200 K/100 μL) or dorsal subcutaneous (S.Q.) implants to the dorsal flank. Because the intraprostate and venous transplant models progressed rapidly and with reproducible kinetics, they provided tumor progression models with quantifiable and predictable survival endpoints (Fig. 1B). To evaluate the effects of WTC dust at different times after exposure, we considered short (3, 7, 21 days), medium (4 months), and long (>1 year) timepoints for our analyses. On the basis of calculated conversion times between mouse and human, these timepoints correlated to about <10, 26, and 58 years, respectively (http://www.age-converter.com/mouse-age-calculator.html). For all experimental cohorts, mice received the standard dosing scheme defined as a 5 mg each day over 4 consecutive days (4 × 5 mg doses) delivered by nasal instillation—an established technique for exposing mice to WTC dust and other environmental agents (17, 18). These approaches have allowed us to model the present-day impact of WTC dust exposure in a manner comparable with what was experienced by first responders on 9/11 (Fig. 1C).

### WTC Dust Components are Retained Primarily in the Lungs After Exposure

We observed WTC dust–exposed lungs, but not PBS controls, to have retention of dark deposits and could be detected for at least 6 months after exposure in all samples examined (low magnification, inset, bar = 100 μm; Fig. 1D). Thus, using the described mouse models, we tested the ability of WTC dust components to distribute and be retained in mouse organs. By mass spectrometry analysis, we assessed a panel of heavy metals known to be present in WTC dust (refs. 1, 3, 31–33; Fig. 1E; Supplementary Fig. S1; *n* = 10). After exposing C57BL/6 mice to WTC dust, we observed the presence of Ti-147, Ti-3, V-251, Sb-121 (where Ti = titanium, V = Vanadium, Sb = Antimony) in lung, spleen, and prostate. However, levels of these metals and others differed dramatically between organs with highest levels present in the lungs whether analyzed at 7 or 21 days after exposure (*n* = 5–8 per organ). For example, Sb-121 was present in the lungs at nearly 1,000× higher levels (mass spectrometry units) than in prostates of the same mice (Supplementary Fig. S1).

Fibrous components including asbestos, talc, and chrysotile with known carcinogenic potential were present in WTC dust (1–3). Using analytic microscopy, we assessed the presence of such components in lung, prostate, thyroid, and fat. In lung, we observed an abundance of WTC dust components, as shown in electron micrographs and companion quantifications using our previously established techniques (34). Fibrous dust components could be detected in lungs from 21 days up to 1 year after exposure including significant retention of chrysotile. Representative images are shown for 21 days after exposure (panels 1+4), tremolite (panels 3), talc (panels 2+6), and aluminum silicate (panels 5–6; Fig. 1F). Fiber counting techniques revealed high fiber counts per wet weight of the lung but no detectable fibers in other organs of the same mice (Fig. 1G, *n* = 7). Thus, metal and fibrous components were retained for prolonged periods and at high levels in the lungs, as compared with other organs in mice exposed to WTC dust by nasal instillation.

### WTC Dust Exposure Induces an Inflammatory Cytokine Gene Signature in Lung and Peripheral Blood

WTC first responders reported events associated with altered pulmonary function including inflammation (16, 35).

**FIGURE 2:**
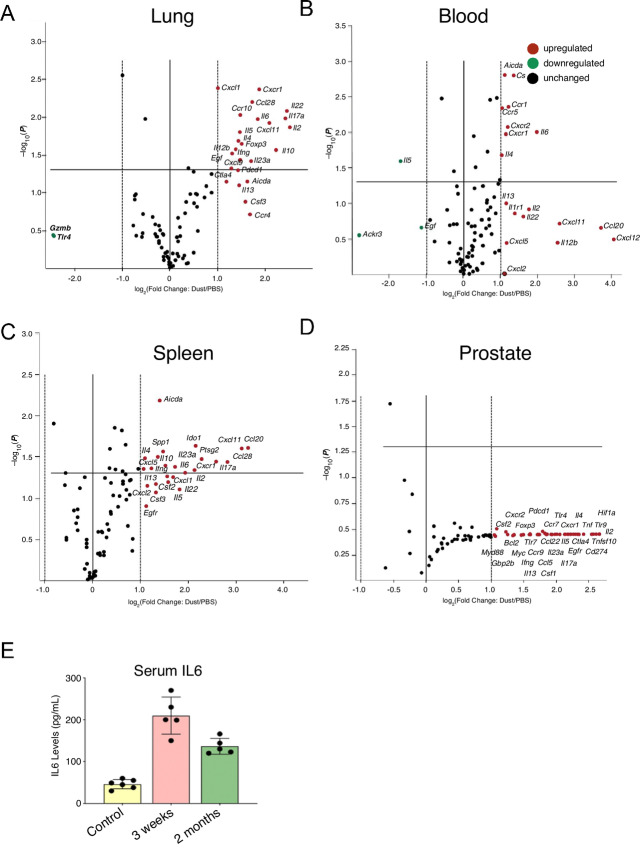
Effect of WTC dust exposure on inflammation and protumor cytokine expression in mouse organs. Volcano plots showing the effects of WTC dust exposure on cytokine gene expression in different organs 21 days after exposure (**A**, lung; **B**, blood; **C**, spleen; **D**, prostate, *n* = 3). The *x* axis for each graph shows gene expressions (in log base 2) while the *y*-axis shows the *P* value for each gene (negative log base 10, Student *t* test of the replicate raw C_T_ data) for each gene on the *y*-axis. Data above the solid horizontal line represents statistically significant values (*P* = 0.05). The solid vertical represents no change in gene expression (log_2_ (1) = 0). Data points to the right of the solid vertical line indicates upregulated genes, and the data points to the left indicate downregulated genes (threshold of 2-fold change). Individual gene expressions are shown as being either upregulated (red), downregulated (green), or unchanged (black). Each graph shows values for WTC dust–exposed mice normalized to PBS-treated controls (>1.0 log_2_ fold change dust/PBS-treated samples). **E,** IL6 levels measured in the sera of mice 21 days (200 pg/mL, *P* < 0.05, *n* = 5) and 2 months (135 pg/mL, *P* < 0.01, *n* = 5) after WTC dust exposure compared with levels in PBS control mouse sera (35 pg/mL, *n* = 6).

We also previously reported the presence of altered gene expression patterns in tumors of patients with prostate cancer who were exposed to WTC dust (36). Thus, to test the hypothesis that WTC dust exposure could initiate an inflammatory signature in men with early disease state (consisting of few PIN lesions and mostly normal glandular structure) we evaluated the effects of WTC dust exposure on inflammatory gene markers in *Wt* prostates at 7 and 21 days after exposure (Fig. 2; Supplementary Fig. S2). For this, mice were exposed to WTC dust followed by resection of lung, prostate, spleen, and blood for RNA-cDNA isolation. Using the RT2 profiler PCR array analysis (Qiagen), we assessed 100 inflammatory-related genes for expression changes. Volcano plots were generated to show the effects of WTC dust exposure on cytokine gene expression in different organs. Plots show the log base 2 for fold change for each gene on the *x*-axis and negative log base 10 for the *P* value (Student *t* test of the replicate raw C_T_^data^) for each gene (*n* = 3) on the *y* axis. Data above the solid horizontal line were statistically significant (*P* = 0.05), whereas the solid vertical line represents no change in gene expression (log_2_ (1) = 0). Data to the right of the solid vertical line indicate upregulated genes, and to those to the left indicate downregulated genes (threshold of 2-fold change). Individual gene expressions are shown as being either upregulated (red), downregulated (green), or unchanged (black). Values for WTC dust–exposed mice were normalized to PBS-treated controls (>1.0 log_2_ fold change dust/PBS-treated samples).

Using this approach, we observed the lungs to have significant (*P* < 0.05) induction of multiple cytokines (*Il2*, *Il4*, *Il5*, *Il6*, *Il12b*, *Il13*), chemokines (*Cxcl1*, *Cxcl9*, *Cxcl11*), and chemokine receptors (*Cxcr1*) 21 days after dust exposure (Table 2). Multiple genes showed greater than 2-fold (log_2_) induction of gene expression including *Il10*, *Il12*, *Il17a,* and *Il22*. In peripheral blood, we also detected significant increases of chemokine receptor genes (*Cxcr1*, *Cxcr2*, *Csf1*) as well as marked induction of *Il6* gene expression. Other noteworthy gene expression increases included *Ifng* (interferon gamma), a cytokine secreted by activated T cells and natural Killer (NK) cells, which can promote macrophage activation and the innate immune system (Fig. 2).

**TABLE 2:** Up–down gene expression values in the lung resulting from WTC dust exposure (normalized to PBS controls).

**Lung (7 day)**		
Gene	*P*	Fold change
*Cxcl5*	0.057	1.83
*Cxcr1*	0.189	1.63
*Bcl2l1*	0.029	1.62
*Cxcr4*	0.0097	−1.32
*Ccr7*	0.0113	−1.32
*Gzma*	0.0083	−1.53
*Il1b*	0.0039	−3.17
**Lung (21 days)**		
**Gene**	***P***	**Fold change**
*Il2*	0.0136	5.75
*Il22*	0.0083	5.49
*Il17a*	0.0104	5.38
*Il10*	0.0274	4.69
*Cxcl11*	0.012	4.25
*Cxcr1*	0.0043	3.68
*Il6*	0.0106	3.6
*Ccl28*	0.0063	3.32
*Il23a*	0.0385	3.27
*Ccr4*	0.1944	3.22
*Aicda*	0.0712	3.1
*Csf3*	0.132	3.02
*Foxp3*	0.0226	2.86
*Ifng*	0.0367	2.81
*Ccr10*	0.0094	2.79
*Il5*	0.0159	2.78
*Il13*	0.0802	2.75
*Il4*	0.0208	2.71
*Pdcd1*	0.0507	2.7
*Il12b*	0.0269	2.61
*Egf*	0.0305	2.48
*Cxcl9*	0.0479	2.45
*Ctla4*	0.0719	2.29
*Cxcl1*	0.0041	2.01
*Tlr4*	0.3751	−5.38
*Gzmb*	0.364	−5.45

The peripheral blood also showed significant induction of cytokines (*Il4*, *Il6*) and chemokine receptors (*Ccr1*, *Ccr5*, *Cxcr1*, *Cxcr5*). Of note, both peripheral blood and spleen revealed the high induction of *Aicda*—a gene known to be involved in chronic inflammation and epigenetic regulation of cancer (37, 38). In the prostate, we observed induction of genes that associate with several signaling pathways; however, these failed to achieve statistical significance. *Together*, these results show that in mice exposed to WTC dust, there is a strong induction of inflammatory and tumor-promoting cytokines in the lung, blood, and spleen (Fig. 2A–C). Among these include *Il6* which has been previously implicated in the promotion of prostate cancer both *in vitro* and *in vivo* (39–43).

### Detection of Elevated IL6 Protein in Peripheral Blood of Mice Exposed to WTC Dust

To determine whether increases in IL6 gene expression in the lung were associated with increases in systemic IL6 protein levels, we isolated sera from control and WTC dust–treated mice. IL6 levels were then assessed by ELISA at two timepoints with the following values: 21 days (200 pg/mL, *P* < 0.005, *n* = 5) and 2 months (135 pg/mL, *P* < 0.001, *n* = 5) after dust exposure. As compared with steady-state levels in control C57BL/6 mice (35 pg/mL, *n* = 6), these values represent marked IL6 increases in the circulation (Fig. 2E).

### WTC Dust Exposure Induces Pulmonary and Prostate Immune Cell Infiltration

**FIGURE 3:**
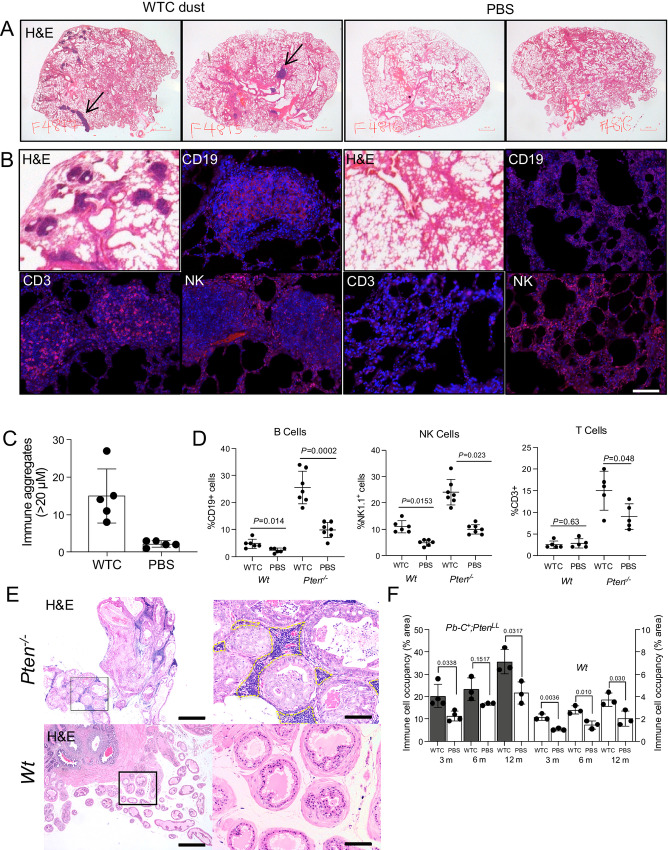
WTC dust exposure promotes immune cell infiltration in the lungs and prostates of normal and GEM models of prostate cancer. **A,** Low magnification H&E images harvested from C57BL/6 mice 3 months after exposure to WTC dust or control PBS control. Significant increases in the presence of lymphoid aggregates are present in the lungs of dust-exposed mice as compared with controls (left bar = 500 μm). **B,** Immune aggregates counted in tissue sections of lungs harvested from WTC (*n* = 5) and PBS (*n* = 5) exposed mice. Aggregates exceeding 20 μm were counted. **C,** Lungs from mice with WTC and PBS treatments showing the accumulation of lymphoid aggregates composed of high numbers of CD19^+^ B cells, CD3^+^ T cell, and NK1.1+ NK cells (black bar = 50 μm, white bar = 100 μm). **D,** Evaluation of immune cell infiltration by flow cytometry in normal and *Pb-Cre^+^;Pten^L/L^* GEM prostates shown for mice 12 weeks after WTC or PBS exposure (*n* = 7–10 per cohort). **E,** Evaluation of immune cell infiltration using Qupath analysis of scanned H&E-stained tissues for WTC- and PBS-treated samples (left bar = 500 μm, right bar = 100 μm). **F,** Measured values for immune cell occupancy Wt and *Pten*-mutant mice exposed at different timepoints shown as a percentage of the total tissue area (bottom).

Lungs isolated from mice exposed to WTC dust showed gross enlargement accompanied by high numbers of immune cell aggregates. Conversely, PBS control lungs showed only occasional immune cell aggregates (WTC = 15.0, PBS = 2.2; *P* = 0.0055, 4 months, *n* = 5 each) and no gross enlargements (Fig. 3A and B, bar = 100 μm). IHC analysis of immune aggregates in lung specimens showed these aggregates composed of CD19^+^ B cells, CD3^+^ T cells, and NK cells (Fig. 3C, bar = 50 μm).

To quantify the immune cell infiltration in the prostates of WTC dust–exposed and PBS-exposed mice, we performed flow cytometry analysis of single-cell dissociates of all prostate lobes. Our results revealed significant increases in WTC dust–exposed mice and control mice including normal and those with mutant PTEN (*Pb-Cre^+^Pten^L/L^)*. Flow analyses showed immune cell percentages as follows: B cells (*Wt*, WTC = 4.8; PBS = 2.3, *P* = 0.014; mutant, WTC = 25.5; PBS = 9.85, *P* = 0.0002), NK cells (*Wt*, WTC = 11.0; PBS = 4.8, *P* = 0.0005; mutant, WTC = 24.0; PBS = 10.0, *P* = 0.0001), CD3^+^ T cells (*Wt*, WTC = 2.5; PBS = 2.8, *P* = 0.63; mutant*,* WTC = 15.0; PBS = 9.0, *P* = 0.041; Fig. 3D). As an independent approach, we measured the infiltrating immune cell content in WTC dust–treated prostates using histologic sections. Relative areas occupied by infiltrating immune cells were determined by digital tissue scans and QuPath software analysis. In PTEN-mutant samples following 3, 6, and 12 months after dust exposure, the immune cell occupancy was significantly increased at the 3-month and 12-month timepoints from PBS controls (3 m, WTC = 20.25, PBS = 11.25, *P* = 0.03; 12 m, WTC = 35.6, PBS = 21.6, *P* = 0.03; Fig. 3E and F, left bar = 500 μm, right bar = 100 μm). Together, these data indicate that mice exposed to WTC exhibited greater immune cell infiltration when compared with PBS control mice and that these differences were detectable both in lung and prostate.

### WTC Dust Exposure Promotes PI3K-AKT Signaling Activation and Prostate Cancer Progression

Epidemiologic data indicate that first responders exposed to WTC dust have higher rates of prostate cancer than unexposed individuals (8, 44, 45, 46). Because most first responders were men that did not have clinically diagnosed prostate cancer, we utilized a GEM model of mouse prostatic neoplasia epithelia (mPIN) which is an early disease state frequently found in undiagnosed individuals (47–49). Thus, using younger *Pb-Cre^+^Pten^L/L^* GEM mice (age 4–6 weeks) and age-matched cre-negative controls, we assessed the impact of WTC dust exposure on early-stage tumor progression including epithelial proliferation, genitourinary (GU) block weight, and changes in epithelial invasion.

**FIGURE 4:**
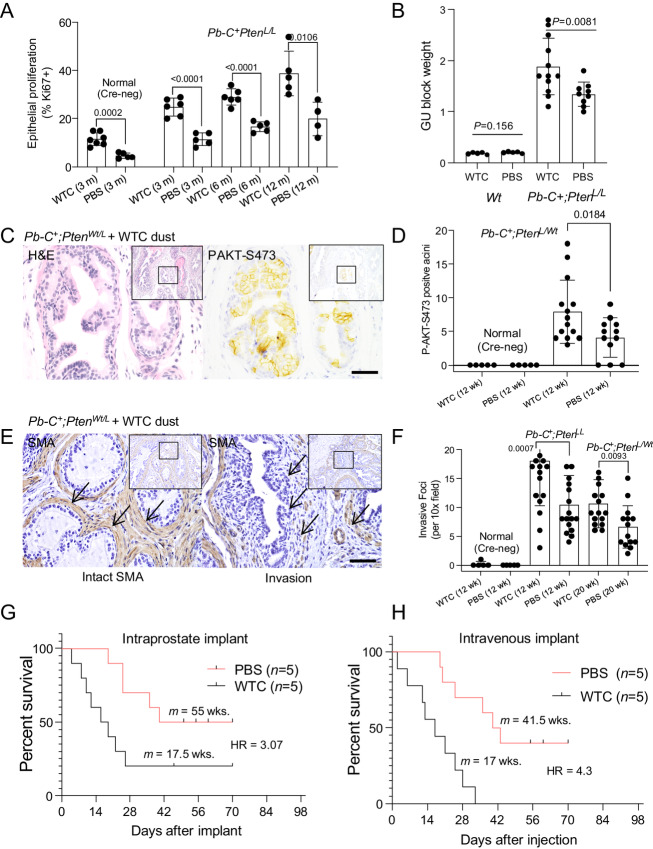
Exposure to WTC dust promotes PI3K-AKT signaling activation and enhanced prostate cancer progression. **A,** WTC dust exposure increases proliferation of the prostatic epithelia. *Wt* (C57BL/6) mice and GEM mice with genetic deletion of both *Pten* alleles (*Pb-Cre^+^;Pten^L/L^*) were exposed to WTC dust at age 6 weeks and evaluated at 3 (*P* < 0.0001), 6 (*P* < 0.0001), and 12 months (*P* < 0.016). Epithelial proliferation (% Ki67^+^ cells) was determined using QuPath software analysis (*n* = 4–7 per timepoint). **B,** WTC dust exposure increases GU block weight (grams) in *Pten*-mutant tumors. Mice with *Wt* (C57BL/6) and *Pten*-deficient prostates were evaluated for changes in GU block weight at 3, 6, and 12 months (aggregate tumor data). **C,** Example of activated AKT expression (P-AKT-S473) in the prostatic acini in *Pb-Cre^+^;Pten^L^^/Wt^*mice (age 12 weeks; bar = 100 μm). **D,** Counted P-AKT-S473–positive acini in WTC dust (*n* = 14 fields) or PBS control (*n* = 11 fields; bar = 100 μm). **E,** Expression of SMA in *Pb-Cre^+^;Pten^L^^/Wt^* mice treated with WTC dust. *Left*, prostatic acini showing continuous SMA distribution and, *right*, an example of discontinuous distribution. **F,** Measures of SMA disruption (epithelial invasion per 10×) in prostatic acini in *Wt* (12 weeks, *n* = 5), *Pb-Cre^+^Pten^L/L^* (12 weeks, *n* = 5) and *Pb-Cre^+^;Pten^L^^/Wt^* (20 weeks, *n* = 5) measured in resected prostates harvested from WTC- and PBS-treated GEM mice. **G,** Effects of WTC dust exposure on C57BL/6 mice 5–7 days after intraprostatic implantation using congenic *Pten* KO tumor cells as compared with PBS-treated controls (2 × 100 K cells surgical implants per prostate; median survival: WTC, 17.5 weeks vs. PBS, 55 weeks, log rank HR = 3.07, *n* = 5). **H,** Effects of WTC dust exposure on C57BL/6 mouse survival with venous injection of *Pten* KO tumor cells (median survival: WTC = 17 weeks, PBS = 41.5 weeks, log-rank HR = 4.3, *n* = 5).

To assess proliferation changes, we stained prostates for Ki67 in *Pb-Cre^+^Pten^L/L^* and cre-negative mice at 3, 6, and 12 m after dust exposure. To enumerate positive cells, stained tissue sections were scanned and counted using the QuPath software. With this approach, we determined increased values for WTC dust–exposed mice relative to PBS controls at all timepoints assessed (Fig. 4A; WTC vs. PBS, 3m, *P* < 0.0001; WTC vs. PBS, 6m, *P* < 0.0001; WTC vs. PBS, 12 m, *P* < 0.0106).

When mice from all timepoints were assessed for differences in total GU block weight, *Wt* or cre-neg prostates showed no difference in WTC-PBS treatment cohorts. However, there were significant weight differences observed between WTC- and PBS-treated mice with PTEN-mutant prostates (*Wt*, *P* = 0.156; mutant, *P* = 0.0081; Fig. 4B). In the 12 m progression cohorts, we observed increased variation in the gross sizes of PTEN-mutant prostates and no significant differences between WTC and PBS cohorts. This observation is consistent with evidence for GU obstruction occurring because of anterior gland expansion of the prostate (30, 50).

During clinical prostate cancer progression, the prostatic epithelium undergoes suppression of PTEN including activation of PI3K-AKT signaling (28). Thus, to model this event in mice and the potential impact of WTC dust exposure, we generated *Pb-Cre^+^;Pten^L^^/Wt^* GEM mice having the loss of a single *Pten* allele. Because environmental agents including carcinogens are known to cooperate with tumor suppressor loss through loss of heterozygosity (51), we considered whether WTC dust exposure could impact the frequency of mPIN lesions in prostatic lesions marked by P-AKT activation. We performed IHC for P-AKT-S473 which is a marker of activated PI3K-AKT signaling occurring in the absence of PTEN protein expression in *Pten* cre-loxp GEM models (14, 30, 52). Quantitation of this marker revealed that *Pb-Cre^+^;Pten^L/Wt^* mice exposed to WTC dust had significantly more P-AKT-S473–positive foci in prostate tissues than PBS-treated *Pb-Cre^+^;Pten^L/Wt^* mice in counts per 10× field (WTC = 7, PBS = 3.3, foci per 10× field, *P* < 0.0184) and in representative IHC immune stains for activated AKT (Fig. 4C and D; Supplementary Fig. S3).

While the PTEN-mutant prostate model does not form significant metastasis, our previous studies have shown that primary tumor epithelial undergo invasion of the stromal compartment (30). Thus, to further investigate whether WTC dust exposure could impact the pathologic progression of primary tumors, we assessed smooth muscle actin (SMA) deposition as an established marker for acinar invasion (14, 30). Prostates with homozygous *Pten* loss showed marked increases in invasive acinar fronts as compared with *Wt* and *Pb-Cre^+^;Pten^L^^/Wt^* mutants. We then counted the number of invasive acini present in WTC dust–treated versus PBS-treated *Pb-Cre^+^;Pten^L^^/L^* mutants (12 weeks) and *Pb-Cre^+^;Pten^L/Wt^* mutants (20 weeks) and observed a significant increase in WTC dust–exposed mice which had more disrupted basement membrane of acini (right, arrows) as compared with PBS-treated mutants which had more intact membranes (left, arrows; Fig. 4E and F). Consistent with our previous studies, we did not detect invasive foci in normal prostatic epithelia. (*Pten^L/L^*; WTC = 18.1; PBS = 10.4 invasive foci; *P* = 0.0007; *Pten^L/Wt^*, WTC = 10.6, PBS = 6.6, *P* = 0.0093; Fig. 4F). Together, these results show that exposure of genetically predisposed prostates to WTC dust promotes elevated PI3K-AKT signaling, increased epithelial proliferation, increased wet weight of the GU block, and increased acinar invasion.

### Impact of WTC Dust Exposure on Progression and Survival in GEM Mice with PTEN-deficient Prostates

We next considered the impact of WTC dust exposure on survival in GEM mice with Pten-deficient autochthonous tumors. Despite the effects on GU tumor size, we did not observe statistically significant differences in mouse survival—a finding potentially related to the fact that Pten-mutant mice rarely die of progressive disease, even with enlarged prostates and symptoms of GU obstruction. Thus, we generated an alternative *in vivo* assay with more reproducible progression kinetics using donor GEM mouse prostates that were resected and digested to the single-cell level. Derived tumor lines were expanded *in vitro* and introduced to C57BL/6 mice either by surgical implantation to the prostate or by single-cell intravenous (tail vein) injection (Figs. 1A and 4G and H). These mice were then exposed to WTC dust or PBS 5–7 days after tumor cell implantation and evaluated for survival. In mice with intraprostate implantation of *Pten* knockout (KO) prostate cells median survival was 17.5 weeks for WTC cohorts and 55 weeks for PBS controls (HR WTC:PBS = 3.62; Fig. 4G). For mice with venous injections, median survival was 17 weeks for WTC cohorts and 41.5 weeks for PBS cohorts (HR WTC:PBS = 7.99; Fig. 4H). Histologic analysis of WTC-treated mice showed lungs to be occupied by markedly more and larger lesions throughout the lungs as compared with PBS controls; however, these lesions presented as being significantly less than those injected with *Pten* KO;*KrasG12D* activated congenic lines. Interestingly, when studies were conducted using S.Q. implants, we detected no significant differences in survival between WTC-PBS treatments as defined by tumors reaching an endpoint of 1.5 cm^2^ IACUC limit (survival, 41 weeks). Together, these data suggest that WTC dust exposure can reduce survival in mice implanted with tumor cells in microenvironments capable of generating a potent inflammatory response.

### Direct Introduction of WTC Dust to the Mouse Prostate Induces Widespread Inflammation

While our analysis supports that most WTC dust components are retained are in the lung, our analysis also revealed the accumulation of low levels of heavy metals in the prostate. Thus, as an exaggerated experimental approach we considered the impact of WTC dust accumulation in the prostates of C57BL/6 mice (Supplementary Fig. S4). To do this, we surgically implanted solubilized WTC dust or control PBS solution directly into the proximal prostate (2× injections, 1–2 mg per prostate lateral lobe; Supplementary Fig. S4A). When the injected prostates were assessed 4–8 weeks after injection, most showed visible retention of dust components appearing as black aggregates (panel 2–4) which were not visible in PBS injected prostates (panel 1). In prostates with injected dust, we also observed marked enlargement of prostates clearly observed at the histologic (Supplementary Fig. S4B and S4D) and gross levels (Supplementary Fig. S4C, panels 2–4). Prostates with dust induced enlargement showed massive infiltration of inflammatory cells and some instances of aberrant histology including increased epithelial proliferation and PIN-like regions (Supplementary Fig. S5). When WTC dust–injected prostates were assessed for changes of inflammatory genes, we used the Qiagen RT2 profiler (WTC, *n* = 3; PBS, *n* = 3) and observed marked induction of genes associated with inflammation. Together, these data show that intraprostate localization of WTC dust is a potent inducer of inflammation and is retained for extended time periods after implantation.

### High-dimensional Analysis of WTC Dust–exposed Patients with Prostate Cancer

**FIGURE 5:**
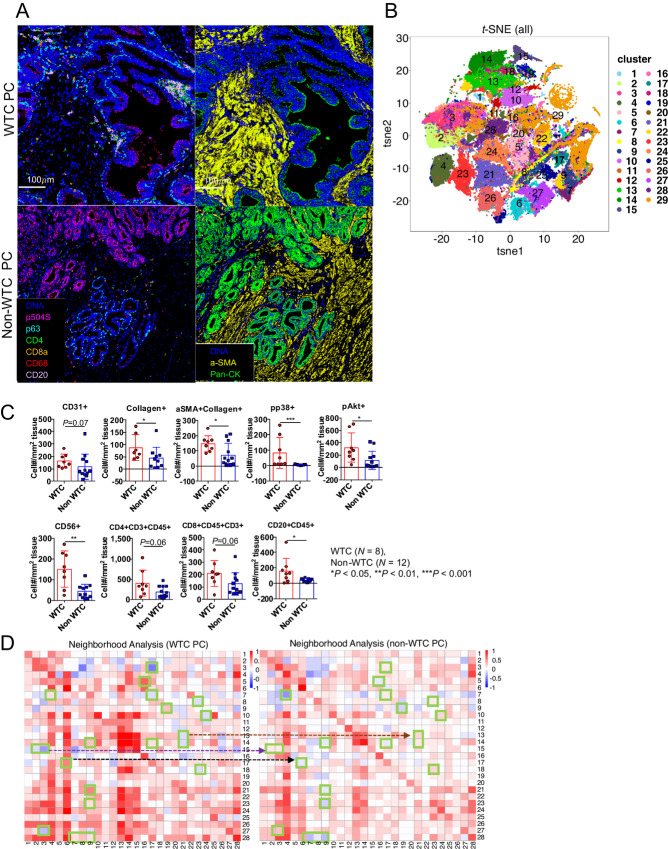
IMC analysis comparing cell clusters in WTC and non-WTC patient prostate tumor tissue. **A,** Representative prostate specimens from WTC patient (*n* = 8) versus non-WTC patients with prostate cancer (*n* = 12) were stained with a panel of 35 markers and shown with selected channels. Scale bar = 100 μm. Aggregate IMC images were processed and clustered into different populations in an unsupervised manner. **B,***t*-SNE plots showing clustering of cell populations from all patients. **C,** Cell density analysis for cell populations. **D,** Neighborhood analysis between WTC and control patients. Boxed populations in neighborhood analysis show examples of significant changes between WTC (*n* = 8) and non-WTC patients (*n* = 12). Mann–Whitney *t* test. *: *P* < 0.05, **: *P* < 0.01, ***: *P* < 0.001 (WTC, *n* = 8 and non-WTC, *n* = 12).

Our mouse modeling studies suggest that WTC dust exposure promotes both inflammation and enhanced prostate cancer progression. To consider whether similar biology could be detected in human tissues, we obtained prostate cancer tissues from WTC survivors and responders with and without WTC dust exposure (WTC, *n* = 8 and non-WTC, *n* = 12). Tissue samples were examined by IMC analysis in a double-blind manner using a 30-marker panel (Table 1) of metal-tagged antibodies and Hyperion ablation (Fluidigm). Patient cell numbers analyzed in IMC analysis is shown for WTC (*n* = 8) and WTC samples (*n* = 12; Supplementary Fig. S6). To address intratumor heterogeneity, we assessed the cell densities (cells per mm^2^) using two independent samplings in 10 patient tumors. Show by meta clusters, we observed minimal variation (Supplementary Fig. 7C). Consistent with our findings in mouse models, we observed increased tissue inflammation and immune cell infiltration in patients with prostate cancer exposed to WTC dust (Fig. 5A). We further clustered and identified the populations by unsupervised algorithms (Fig. 5B; Supplementary Fig. S8). We found a significant increase of αSMA+collagen+ cells (*P* = 0.03, cluster 9) in WTC dust–exposed patients (Fig. 5C; Supplementary Fig. S7), data that are compatible with increased acinar invasion in homozygous *Pten* KO mice (Fig. 4E and F).

Moreover, immune cell infiltration was elevated in WTC dust–exposed patients (Fig. 5C) including NK cell (*P* = 0.009, cluster 17), CD4 T cell (*P* = 0.06, cluster 13), CD8 T cell (*P* = 0.06, cluster 14), and B cells (*P* = 0.03, cluster 15). In addition, WTC dust–exposed patients showed a significant accumulation of phospho-p38+ cells (*P* = 0.0009, cluster 20) and phospho-AKT+ cells (*P* = 0.016, cluster 26), further suggesting a more inflamed tumor environment. Using aggregate IMC datasets of WTC and non-WTC cohorts, we performed neighborhood analysis to reveal the spatial relationship between these identified populations (Fig. 5D). We detected distinct spatial patterns between WTC dust–exposed patients and non-WTC patient groups (green boxes, Fig. 5D). For example, despite increased number of B cells found in WTC patients, these cells are dispersed away from tumor cells (p504S+PanCK+ cells, cluster 2) and epithelial cells (PanCK+ cells, cluster 3). Conversely, the B cells in non-WTC patients with prostate cancer are in neighbored with tumor cells and epithelial cells (purple dash arrow, Fig. 5D). These data suggest that B cells in WTC patients might have different immunologic functions. In addition, NK cells (cluster 17) are proximal with blood vessels (cluster 6) in WTC patients but not in non-WTC patients (black dash arrow, Fig. 5D), reflecting their increased numbers. Finally, the distribution of CD4 and CD8 T cells suggests an avoidance of antigen-presenting cells (HLA-DR+, cluster 21) in WTC patients, but not in non-WTC patients (brown dash arrow, Fig. 5D). These data suggests that T cells in WTC prostate tissue are not well primed and unable to eliminate tumor cells. To show a visual representation of data from Fig. 5, we mapped select clusters to create pseudoimages for WTC and non-WTC cohorts (Supplementary Fig. S8). Together, these data suggest an inflamed tumor environment in WTC-exposed patients with prostate cancer with aberrant immune cell function.

## Discussion

The destruction of the World Trade Towers during the 9/11 attacks resulted in the emission of materials that are implicated in promoting both inflammation and cancer. Epidemiologic studies have established that prostate cancer is among the few organs, to date, demonstrating above background levels of cancer incidence (53, 54). Despite these observational studies, a causal association between WTC dust exposure and changes in prostate cancer progression has not been demonstrated.

In this study, we used well-defined genetically engineered mouse models of prostate cancer to test the hypothesis that acute exposure to WTC dust can promote pathogenic changes to the prostate. Our hypothesis is based on the supposition that WTC first responders, who have since been diagnosed with prostate cancer, contained prostate cancer precursor lesions or very early-staged cancer which were accelerated by the effects of the dust.

To model this hypothesis, we elected to use a GEM model with conditional loss of a single or both Pten alleles resulting in phenotypes that include low-high grade PIN and progression to invasive prostate cancer. With this, our study demonstrated that exposure to WTC dust resulted in: (i) the high accumulation of metals and fibers in the lung but low retention of these dust components in the prostate, (ii) significant acute and chronic inflammatory gene expression changes in the lung and peripheral blood, and (iii) increased proliferation of the normal prostatic epithelia but without the induction of precancer or cancer phenotypes. Conversely, WTC dust led to (iv) accelerated tumorigenesis in prostate epithelia having heterozygous and homozygous deletion of the *Pten* tumor suppressor mediate by increased incidence of lesions with activated PI3K-AKT signaling. (v) We further evaluated the inflammatory and immune cell profile of prostate tumor tissues from the WTC dust–exposed patients by IMC. Increases in the immune cell infiltration, stromal tissue fibrosis and inflammation gene expression were consistently observed in WTC patient cohorts as compared with non-WTC patients with prostate cancer. Results are in line with our previously published NanoString data (36) and methylation studies (55). Together, these findings support the hypothesis that WTC dust causes the production of tumor-promoting cytokines in the lung, which circulate, and then act to increase epithelial proliferation and tumor progression in organs having a predisposition for cancer progression.

Epidemiologic data from independent investigators have indicated that prostate cancer and thyroid cancers have been diagnosed at higher rates in WTC responders as compared with cancer registry data (4–7). These data raise the question about why other organ systems, such as the lung, have not presented with increased cancer rates. One possible explanation is the prevalence of predisposing genetic lesions found in populations that go on to develop prostate cancer or thyroid cancer. Currently, it is unclear whether WTC responders had such similar lesion in their lungs.

The median age for WTC first responders on September 11, 2001, was 50.0 (0.4) years (56), an age at which clinically diagnosed prostate cancer is rare as compared with men 60–80 years of age who have a 1 in 6 chance of cancer detection. Despite this, in the age range of 30–40 years up to 29% of men present with high-grade PIN lesions and microscopic carcinomas indicating that PIN/low-grade cancers can occur well before the typical clinical diagnosis of prostate cancer (57). However, with the long-term retention of cancer promoting fibers in the lungs of WTC responders, it is conceivable that lung cancers and other pulmonary conditions will develop in time as the lung acquires genetic alterations that are cooperative with dust-induced inflammation.

The PTEN tumor suppressor is mutated, deleted, or expressed at reduced levels in a high percentage of human prostate cancers (28, 29) including *PTEN* loss of heterozygosity in up to 20% of localized prostate cancers and more than 60% in advanced disease states. With this information, the Pten conditional GEM model served as a useful modeling approach to address our hypothesis in which we made several striking observations including the induction of inflammatory cytokines in the lung and blood. Of interest, are those cytokines with roles in tumor progression, tumor inhibition as well as the immune system.

In our gene expression analysis, we identified significant differential expression of such cytokines in the lung (*Il2*, *Il4*, *Il10*, Il6, *Il12b*, *Il17a*, *Il22,* and *Il23a*) as well as the peripheral blood (*Il4*, *Il6*). IL6 is a cytokine with well-established protumor function mediated principally by the activation of JAKs and induction of STAT3 at target genes by tyrosine phosphorylation. In prostate cancer, multiple lines of evidence support IL6 to be a driver of progression. For example, in cell line models of prostate cancer, overexpression of IL6 enhances cellular proliferation, tumor progression, and neuroendocrine differentiation (39–43). In the TRAMP genetic mouse model of prostate cancer, epithelial deletion of IL6 functions delays progression (41). In clinical samples, serum IL6 relates both with tumor initiation and progression (58).

Our study has several limitations. While the PTEN GEM model of prostate cancer mimics many aspects of human disease progression, this single-hit genetic model does not fully recapitulate the genetic multifocality often observed in human disease. Thus, while our preclinical modeling supports a role for WTC dust–induced PI3K-AKT activation, it is unclear whether the same mechanism leads to the increased prostate cancer progression observed in human WTC cohorts. This is underscored by the fact that other factors such as receptor tyrosine kinase signaling and promoter methylation can lead to PTEN loss and the resulting activation of PI3K-AKT signaling (59). In addition, while IMC analysis provides a high level of marker expression data, the nature of this technique limited our ability to assess the entire tumor. As a result, the significant heterogeneity often observed in human prostate cancers cannot be fully accounted for. Future analyses should resolve strategies that can fully account for the impact that exposure to WTC dust, and other environmental agents, has through the entirety of primary prostate cancer tumors.

Previous investigations have demonstrated that asbestos and other fibrous like structures found in WTC dust can persist in the lungs for decades resulting in a chronic oxidative stress and inflammation (53, 54). Our analytic microscopy data are consistent with these findings. Thus, it is plausible that as mutations and genetic alterations are acquired through time, a greater number and more diverse spectrum of cancer types will be detected. If systemic levels of protumor cytokines, such as IL6, are shown to be chronically elevated in those exposed to WTC dust, more frequent monitoring for cancer biomarkers such as PSA may be advisable to facilitate early detection of disease. If true, these data warrant further investigations into the impact of highly expressed of pro tumor cytokines on organ systems in individuals exposed to WTC dust nearly 20 years ago. Furthermore, for those responders with early diagnosed prostate cancer or family history, the use of small molecule inhibitors or blocking antibodies may be an appropriate therapeutic course.

## Supplementary Material

Fig S1 Fig. S1. Percentage increases of metals detected by mass spectrometry in lung (A), spleen (B) and prostate (C) found in mice receiving WTC dust normalized to control, PBS nasal instillation (n=8 per organ).

Fig S2 Fig. S2. Volcano plots showing gene expression profiles for C57BL/6 mouse organs at 7d and 21d after WTC dust exposure normalized to PBS control mice (n=3).

Fig S3 Fig. S3. A-B, Expression of P-AKT-S473 in the prostatic acini from WTC dust treated Pb-Cre+PtenL/Wt mice shown at low and high magnifications (A = early progression, B = later progression). C, P-AKT-S473 expression in Pb-Cre+PtenL/L GEM mice (HE bar = 250 μM, IHC bar = 100 μm). D, IF expression of PTEN and P-AKTS473 in Pb-Cre+PtenL/Wt GEM mice treated with WTC dust.

Fig S4 Fig. S4. Effect of direct introduction of WTC dust to the mouse prostate. A, Approach injecting solubilized WTC dust (2x 1 μl injections per prostate) and evaluation 6-8 weeks later. B, Histological low magnification image showing prostate enlargement after dust injection. C, Gross images showing a PBS control injected prostate (panel 1) and prostates with WTC dust injection (panels 2, 3, 4). D, Histological high magnification view showing immune cell infiltration in WTC dust injected prostate. E, Gene expression profiles for two independent prostates injected with WTC dust normalized to PBS control prostates (bars: B, C1, C3 = 2000 μM, C2, C4 = 5000 μM, D = 250 μM).

Fig S5 Fig. S5. Effect of WTC dust injection on prostate histology and epithelial proliferation. A, whole prostate scan showing widespread stromal and immune cell expansion. B, Low and high magnification fields showing AR positive prostate acini with increased Ki67 expression (lower mag bar = 250 μM, higher mag bar 100 μM)

Fig S6 Fig. S6. Cell densities of different cell populations in WTC and non-WTC patient tissues samples. A, Immunoreactive cells for individual antibodies tested in WTC and non-WTC IMC analysis (WTC, n=8 patient samples and non-WTC, n=12 patient samples). Graphs were generated based on the analysis of tSNE plots shown in Fig. S7. B, Cell numbers used for IMC analysis for non-WTC and WTC analysis

Fig S7 Fig. S7. Aggregate tSNE plots generated from aggregate analysis of different clusters of WTC and control patients. A, Circled populations in t-SNE plots showed significant differences between WTC and control patients. B, List of markers used to define the clusters identified in tSNE plots (WTC, n=8 and non-WTC, n=12). C, Meta clusters showing the difference in cell density values occurring in IMC tissue samplings (n=2) for each patient sample (n=10).

Fig S8 Fig. S8. Pseudo images showing the spatial distribution between representative clusters in WTC and non-WTC human prostate cancer cohorts.
