# Supplementary material for: World Trade Center Dust Exposure Promotes Cancer in PTEN-deficient Mouse Prostates
Source: Cancer Res Commun. 2022 Jun 27;2(6):518–32. doi: 10.1158/2767-9764.CRC-21-0111 (PMC9336209; doi:10.1158/2767-9764.CRC-21-0111)
Supplement: Fig S1 — Fig. S1. Percentage increases of metals detected by mass spectrometry in lung (A), spleen (B) and prostate (C) found in mice receiving WTC dust normalized to control, PBS nasal instillation (n=8 per organ). [file crc-21-0111-s01.pdf]

Fig. S1

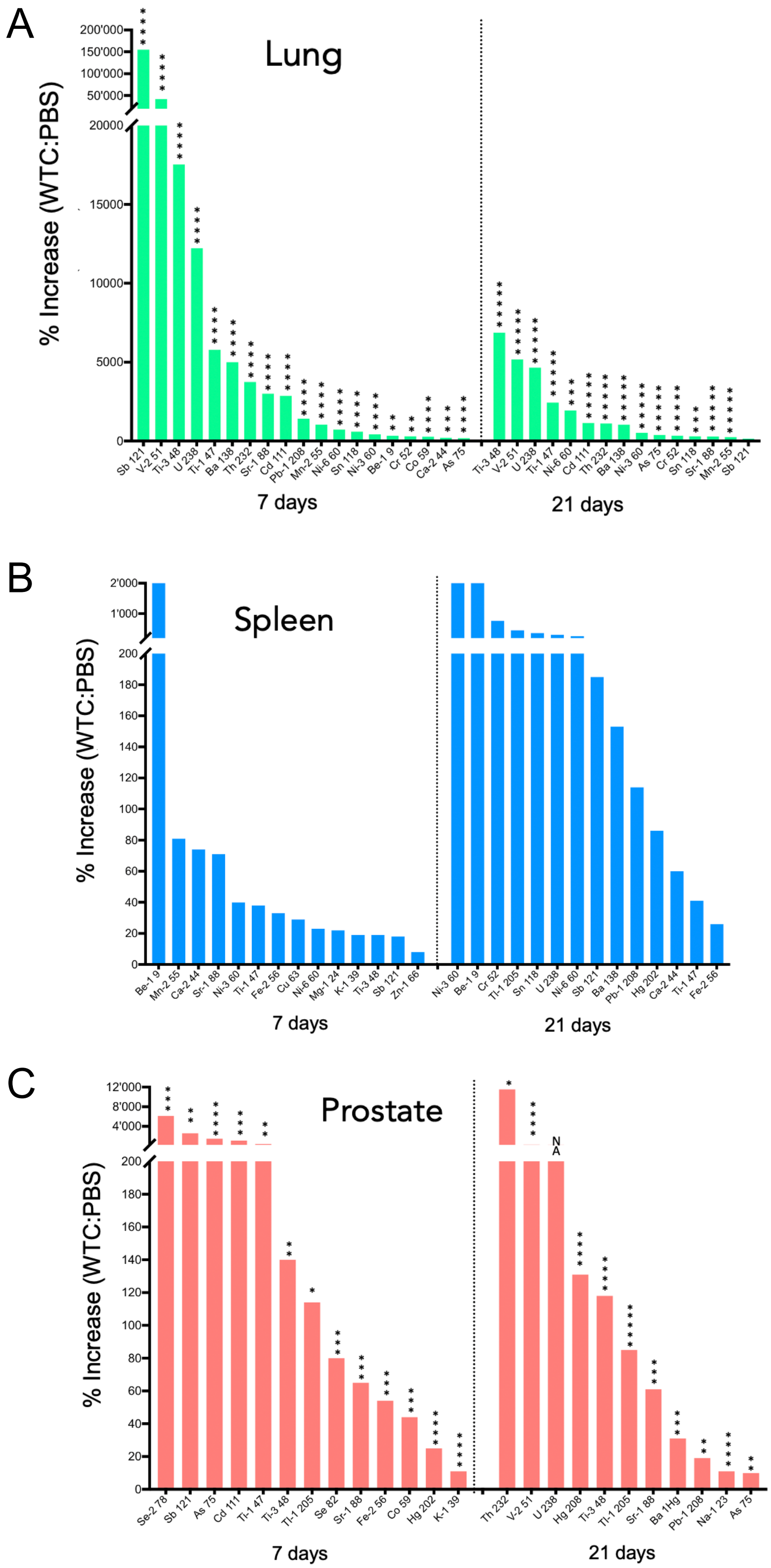
A
Lung
% Increase (WTC:PBS)
7 days
21 days
B
Spleen
% Increase (WTC:PBS)
7 days
21 days
C
Prostate
% Increase (WTC:PBS)
7 days
21 days

Fig. S1. Percentage increases of metals detected by mass spectrometry in lung (A), spleen (B) and prostate (C) found in mice receiving WTC dust normalized to control, PBS nasal instillation (n=8 per organ).
