# Supplementary material for: World Trade Center Dust Exposure Promotes Cancer in PTEN-deficient Mouse Prostates
Source: Cancer Res Commun. 2022 Jun 27;2(6):518–32. doi: 10.1158/2767-9764.CRC-21-0111 (PMC9336209; doi:10.1158/2767-9764.CRC-21-0111)
Supplement: Fig S2 — Fig. S2. Volcano plots showing gene expression profiles for C57BL/6 mouse organs at 7d and 21d after WTC dust exposure normalized to PBS control mice (n=3). [file crc-21-0111-s02.pdf]

Fig. S2

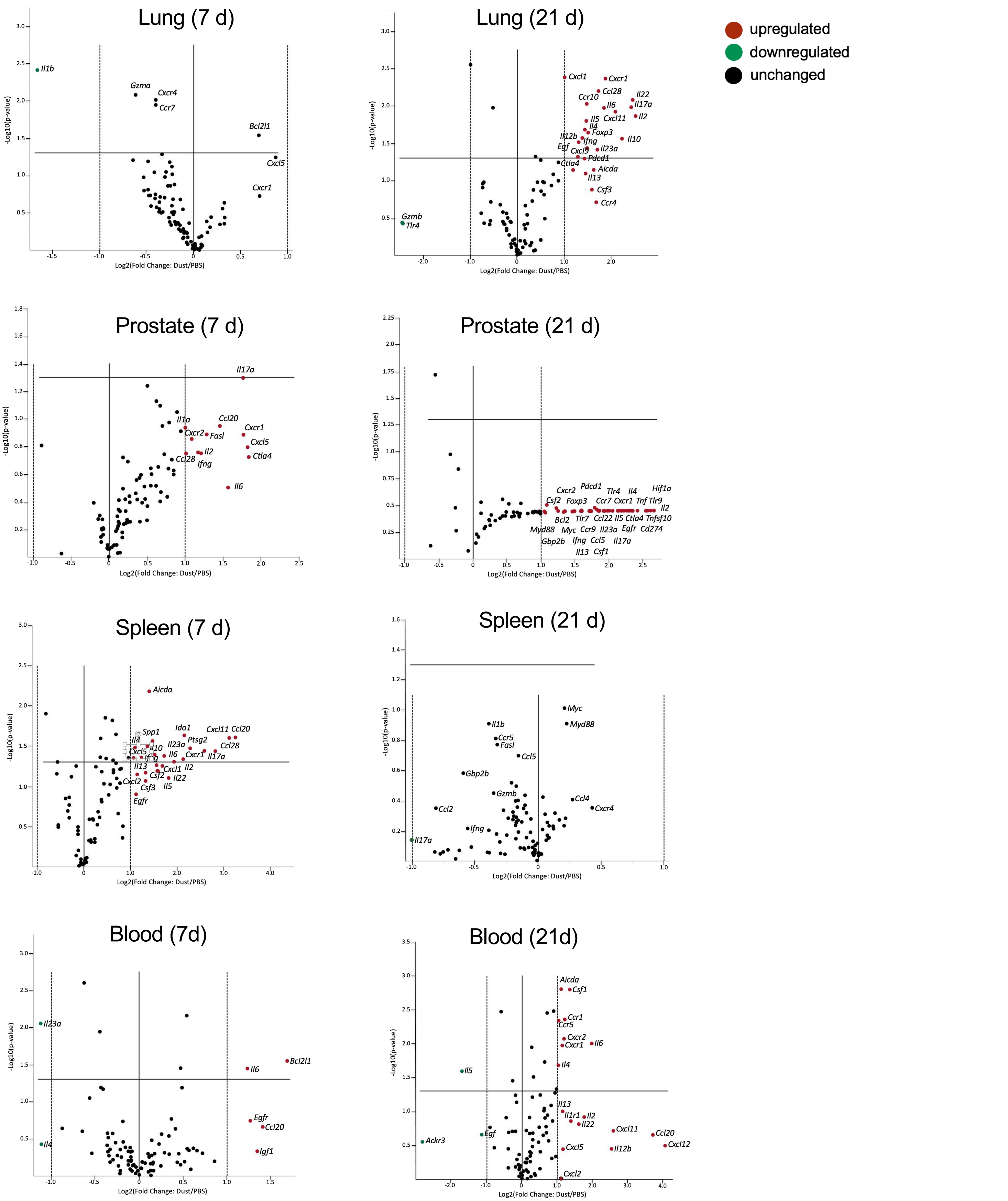
upregulated
downregulated
unchanged
Lung (7 d)
-Log10(p-value)
Log2(Fold Change: Dust/PBS)
Il1b
Gzma
Cxcr4
Ccr7
Bcl2l1
Cxcl5
Cxcr1
Lung (21 d)
Cxcl1
Cxcr1
Ccl28
Ccr10
Il22
Il17a
Il6
Il5
Cxcl11
Il2
Il4
Foxp3
Il12b
Ifng
Il10
Egf
Cxcl9
Il23a
Pdcd1
Ctla4
Aicda
Il13
Csf3
Ccr4
Gzmb
Tlr4
Prostate (7 d)
Il17a
Ccl20
Il1a
Cxcr1
Cxcr2
Fasl
Cxcl5
Il2
Ctla4
Ccl28
Ifng
Il6
Prostate (21 d)
Cxcr2
Pdcd1
Tlr4
Il4
Hif1a
Csf2
Foxp3
Ccr7
Cxcr1
Tnf
Tlr9
Il2
Bcl2
Tlr7
Ccl22
Il5
Ctla4
Tnfsf10
Myd88
Myc
Ccr9
Il23a
Egfr
Cd274
Gbp2b
Ifng
Ccl5
Il17a
Il13
Csf1
Spleen (7 d)
Aicda
Spp1
Ido1
Cxcl11
Ccl20
Il4
Il23a
Ptsg2
Ccl28
Cxcl5
Il10
Ifng
Il6
Cxcr1
Il17a
Il13
Cxcl1
Il2
Cxcl2
Csf2
Il22
Csf3
Il5
Egfr
Spleen (21 d)
Myc
Myd88
Il1b
Ccr5
Fasl
Ccl5
Gbp2b
Gzmb
Ccl4
Ccl2
Cxcr4
Ifng
Il17a
Blood (7d)
Il23a
Bcl2l1
Il6
Egfr
Ccl20
Il4
Igf1
Blood (21d)
Aicda
Csf1
Ccr1
Ccr5
Cxcr2
Il6
Cxcr1
Il4
Il5
Il13
Il1r1
Il2
Il22
Cxcl11
Ccl20
Egf
Ackr3
Cxcl5
Il12b
Cxcl12
Cxcl2

Fig. S2. Volcano plots showing gene expression profiles for C57BL/6 mouse organs at 7d and 21d after WTC dust exposure normalized to PBS control mice (n=3).
