# Supplementary material for: World Trade Center Dust Exposure Promotes Cancer in PTEN-deficient Mouse Prostates
Source: Cancer Res Commun. 2022 Jun 27;2(6):518–32. doi: 10.1158/2767-9764.CRC-21-0111 (PMC9336209; doi:10.1158/2767-9764.CRC-21-0111)
Supplement: Fig S3 — Fig. S3. A-B, Expression of P-AKT-S473 in the prostatic acini from WTC dust treated Pb-Cre+PtenL/Wt mice shown at low and high magnifications (A = early progression, B = later progression). C, P-AKT-S473 expression in Pb-Cre+PtenL/L GEM mice (HE bar = 250 μM, IHC bar = 100 μm). D, IF expression of PTEN and P-AKTS473 in Pb-Cre+PtenL/Wt GEM mice treated with WTC dust. [file crc-21-0111-s03.pdf]

Fig. S3

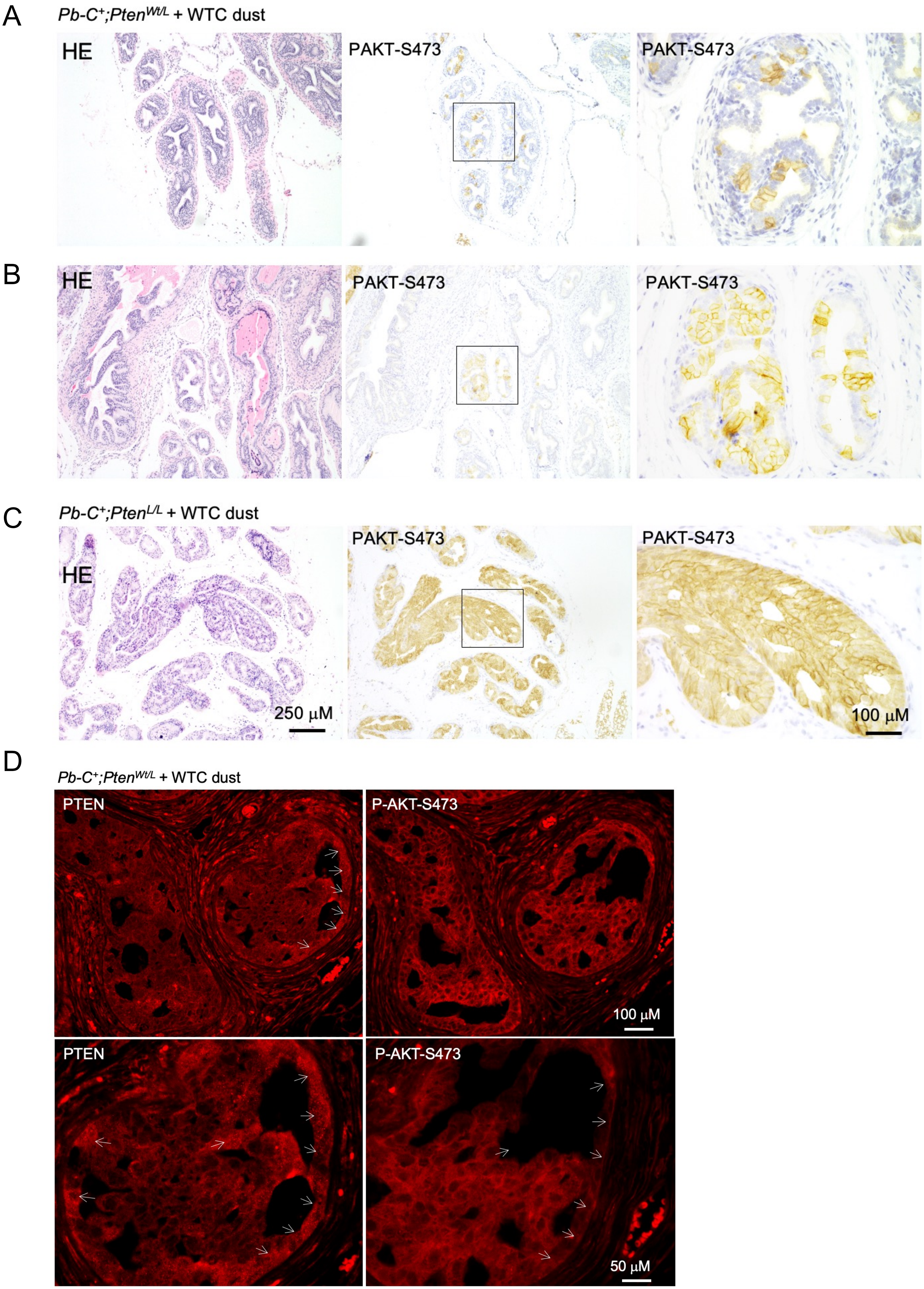

A
Pb-C+;PtenWt/L + WTC dust
HE
PAKT-S473
PAKT-S473
B
HE
PAKT-S473
PAKT-S473
C
Pb-C+;PtenL/L + WTC dust
HE
PAKT-S473
PAKT-S473
250 μM
100 μM
D
Pb-C+;PtenWt/L + WTC dust
PTEN
P-AKT-S473
100 μM
PTEN
P-AKT-S473
50 μM

Fig. S3. A-B, Expression of P-AKT-S473 in the prostatic acini from WTC dust treated *Pb-Cre*$^{+}$;*Pten*$^{L/Wt}$ mice shown at low and high magnifications (A = early progression, B = later progression). **C**, P-AKT-S473 expression in *Pb-Cre*$^{+}$;*Pten*$^{L/L}$ GEM mice (HE bar = 250 μM, IHC bar = 100 μM). **D**, IF expression of PTEN and P-AKT-S473 in *Pb-Cre*$^{+}$; *Pten*$^{L/Wt}$ GEM mice treated with WTC dust.
