# Supplementary material for: World Trade Center Dust Exposure Promotes Cancer in PTEN-deficient Mouse Prostates
Source: Cancer Res Commun. 2022 Jun 27;2(6):518–32. doi: 10.1158/2767-9764.CRC-21-0111 (PMC9336209; doi:10.1158/2767-9764.CRC-21-0111)
Supplement: Fig S4 — Fig. S4. Effect of direct introduction of WTC dust to the mouse prostate. A, Approach injecting solubilized WTC dust (2x 1 μl injections per prostate) and evaluation 6-8 weeks later. B, Histological low magnification image showing prostate enlargement after dust injection. C, Gross images showing a PBS control injected prostate (panel 1) and prostates with WTC dust injection (panels 2, 3, 4). D, Histological high magnification view showing immune cell infiltration in WTC dust injected prostate. E, Gene expression profiles for two independent prostates injected with WTC dust normalized to PBS control prostates (bars: B, C1, C3 = 2000 μM, C2, C4 = 5000 μM, D = 250 μM). [file crc-21-0111-s04.pdf]

Fig. S4

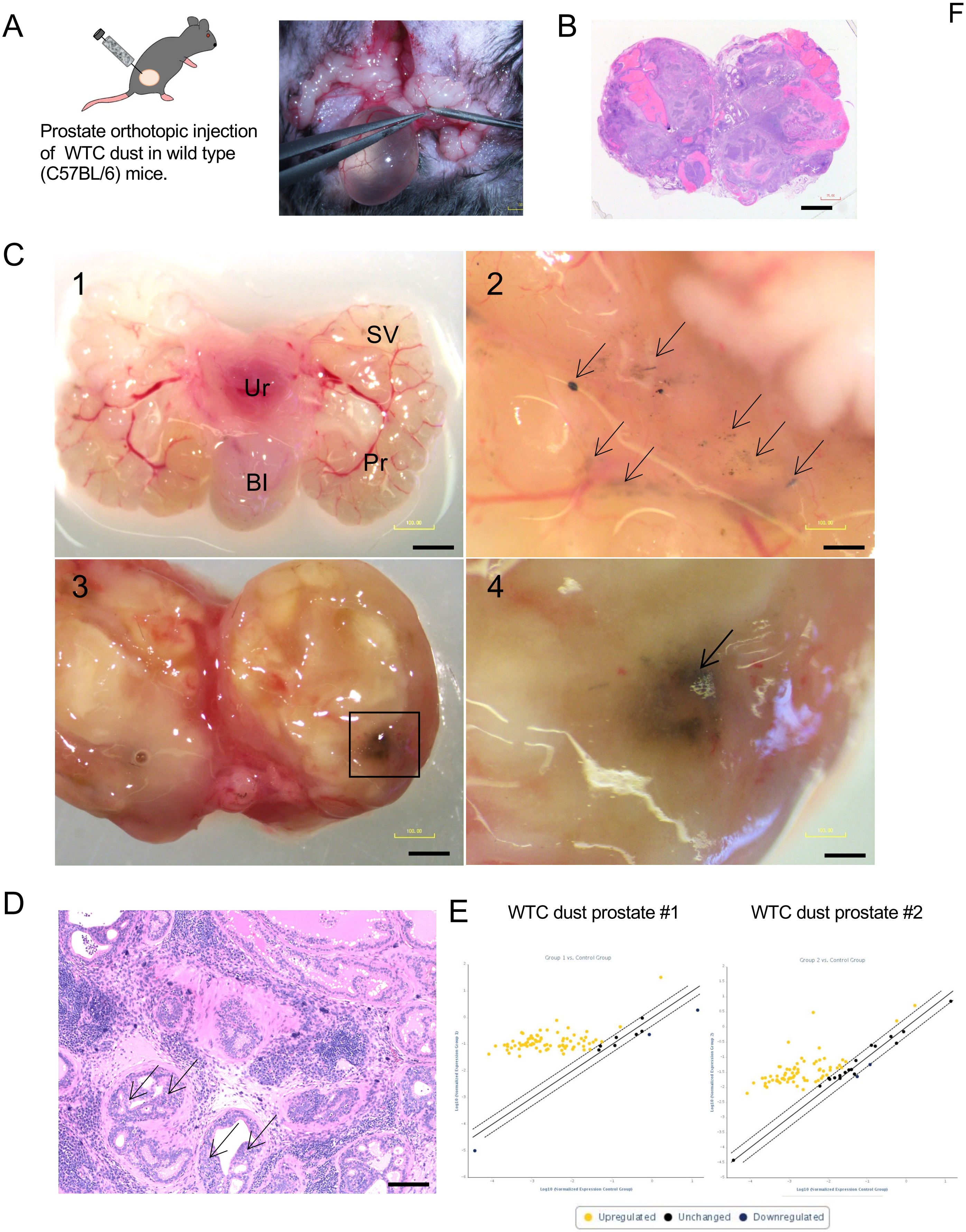
A
Prostate orthotopic injection of WTC dust in wild type (C57BL/6) mice.
B
C
1
SV
Ur
Bl
Pr
2
3
4
D
E
WTC dust prostate #1
WTC dust prostate #2
Group 1 vs. Control Group
Group 2 vs. Control Group
Upregulated
Unchanged
Downregulated

Fig. S4. Effect of direct introduction of WTC dust to the mouse prostate. **A**, Approach injecting solubilized WTC dust (2x 1 μl injections per prostate) and evaluation 6-8 weeks later. **B**, Histological low magnification image showing prostate enlargement after dust injection. **C**, Gross images showing a PBS control injected prostate (panel 1) and prostates with WTC dust injection (panels 2, 3, 4). **D**, Histological high magnification view showing immune cell infiltration in WTC dust injected prostate. **E**, Gene expression profiles for two independent prostates injected with WTC dust normalized to PBS control prostates (bars: B, C1, C3 = 2000 μM, C2, C4 = 5000 μM, D= 250 μM).
