# Supplementary material for: World Trade Center Dust Exposure Promotes Cancer in PTEN-deficient Mouse Prostates
Source: Cancer Res Commun. 2022 Jun 27;2(6):518–32. doi: 10.1158/2767-9764.CRC-21-0111 (PMC9336209; doi:10.1158/2767-9764.CRC-21-0111)
Supplement: Fig S5 — Fig. S5. Effect of WTC dust injection on prostate histology and epithelial proliferation. A, whole prostate scan showing widespread stromal and immune cell expansion. B, Low and high magnification fields showing AR positive prostate acini with increased Ki67 expression (lower mag bar = 250 μM, higher mag bar 100 μM) [file crc-21-0111-s05.pdf]

Fig. S5

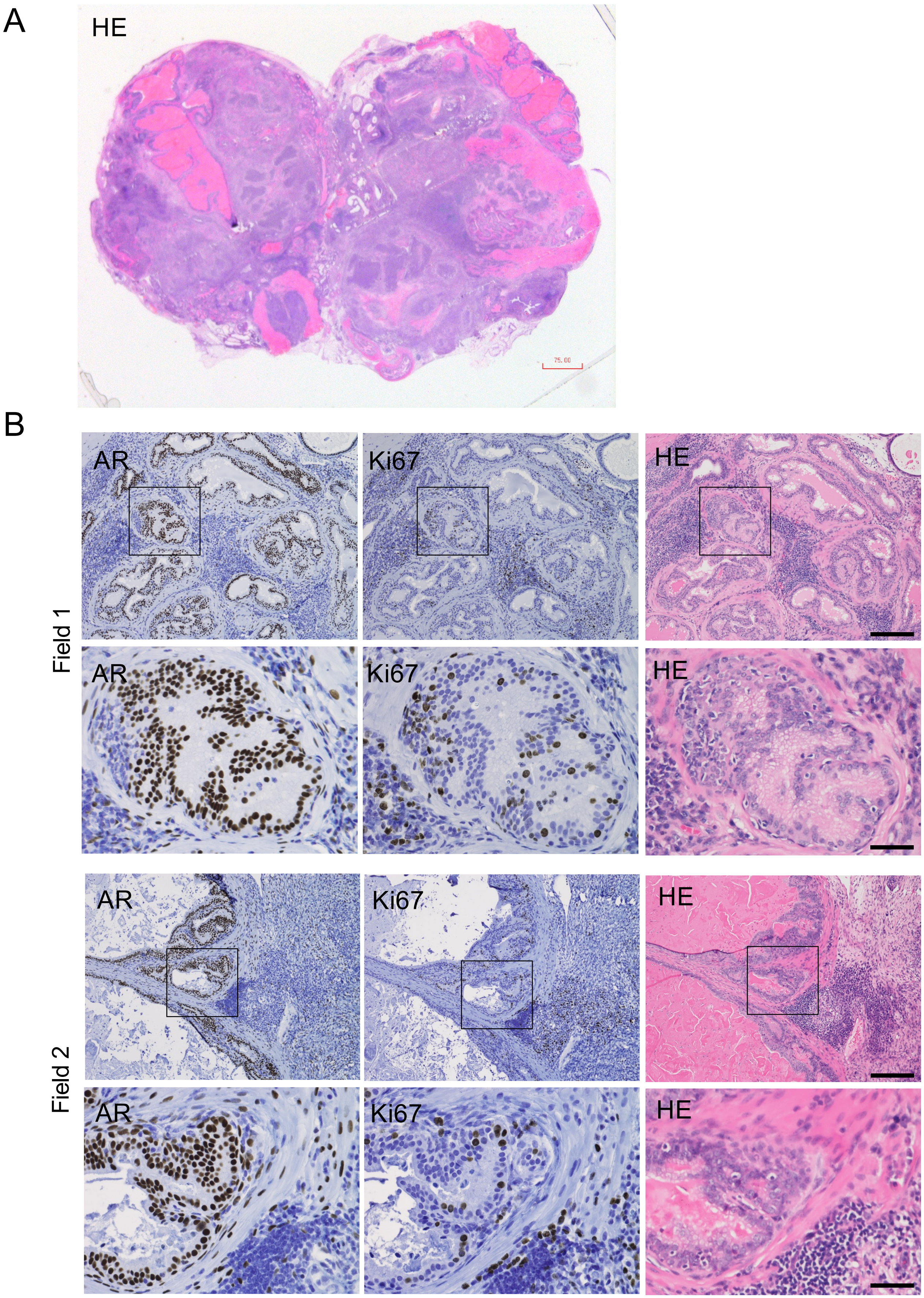

Fig. S5. Effect of WTC dust injection on prostate histology and epithelial proliferation. **A**, whole prostate scan showing widespread stromal and immune cell expansion. **B**, Low and high magnification fields showing AR positive prostate acini with increased Ki67 expression (lower mag bar = 250 μM, higher mag bar 100 μM).
