# Supplementary material for: World Trade Center Dust Exposure Promotes Cancer in PTEN-deficient Mouse Prostates
Source: Cancer Res Commun. 2022 Jun 27;2(6):518–32. doi: 10.1158/2767-9764.CRC-21-0111 (PMC9336209; doi:10.1158/2767-9764.CRC-21-0111)
Supplement: Fig S6 — Fig. S6. Cell densities of different cell populations in WTC and non-WTC patient tissues samples. A, Immunoreactive cells for individual antibodies tested in WTC and non-WTC IMC analysis (WTC, n=8 patient samples and non-WTC, n=12 patient samples). Graphs were generated based on the analysis of tSNE plots shown in Fig. S7. B, Cell numbers used for IMC analysis for non-WTC and WTC analysis [file crc-21-0111-s06.pdf]

Fig. S6

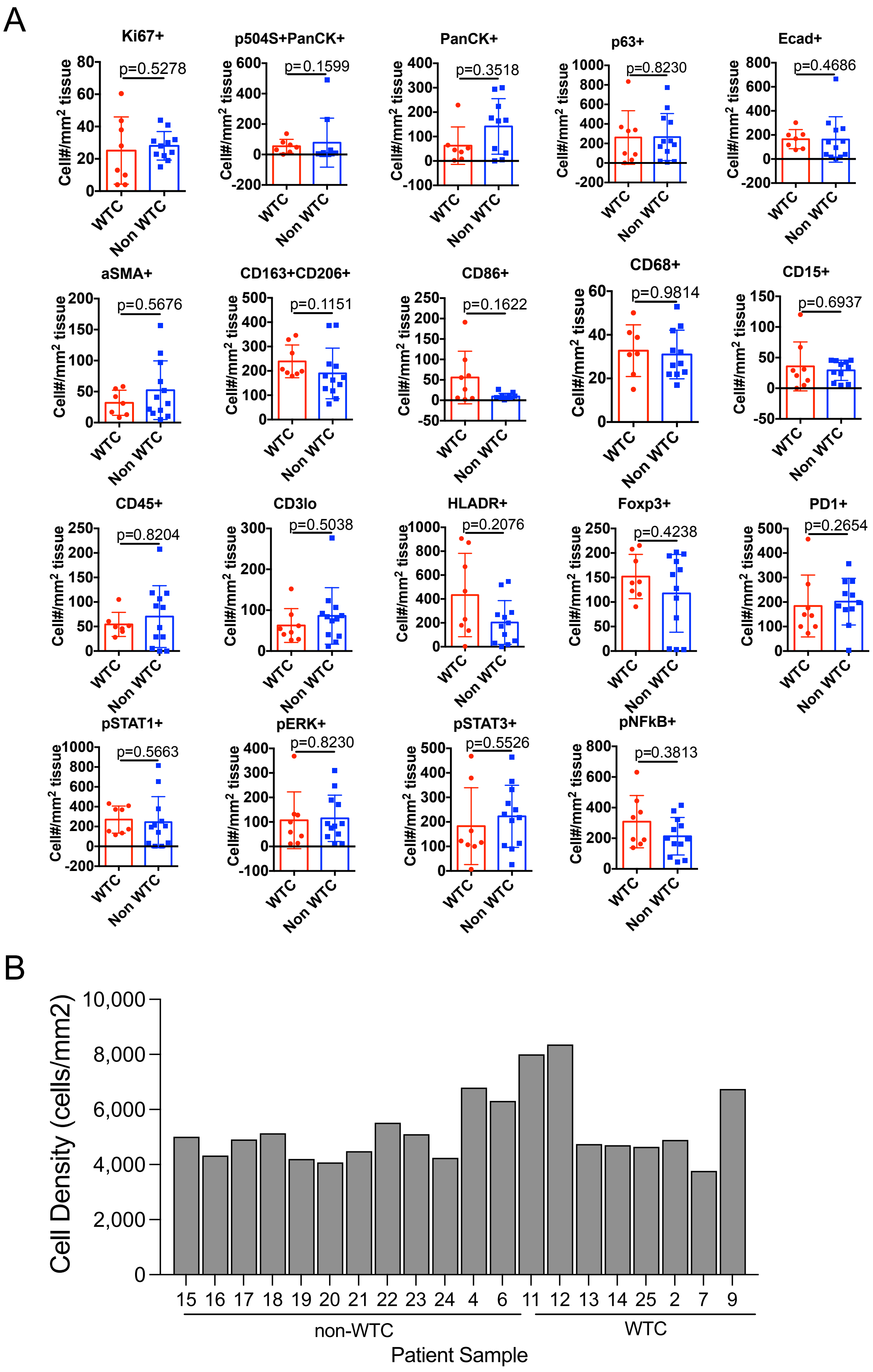
A
Ki67+
p=0.5278
p504S+PanCK+
p=0.1599
PanCK+
p=0.3518
p63+
p=0.8230
Ecad+
p=0.4686
aSMA+
p=0.5676
CD163+CD206+
p=0.1151
CD86+
p=0.1622
CD68+
p=0.9814
CD15+
p=0.6937
CD45+
p=0.8204
CD3lo
p=0.5038
HLADR+
p=0.2076
Foxp3+
p=0.4238
PD1+
p=0.2654
pSTAT1+
p=0.5663
pERK+
p=0.8230
pSTAT3+
p=0.5526
pNFkB+
p=0.3813
Cell#/mm2 tissue
WTC
Non WTC
B
Cell Density (cells/mm2)
10,000
8,000
6,000
4,000
2,000
0
15 16 17 18 19 20 21 22 23 24 4 6 11 12 13 14 25 2 7 9
non-WTC
WTC
Patient Sample

Fig. S6. Cell densities of different cell populations in WTC and non-WTC patient tissues samples.

A, Immunoreactive cells for individual antibodies tested in WTC and non-WTC IMC analysis (WTC, n=8 patient samples and non-WTC, n=12 patient samples). Graphs were generated based on the analysis of tSNE plots shown in Fig. S7. **B**, Cell numbers used for IMC analysis for non-WTC and WTC analysis.
