# Supplementary material for: World Trade Center Dust Exposure Promotes Cancer in PTEN-deficient Mouse Prostates
Source: Cancer Res Commun. 2022 Jun 27;2(6):518–32. doi: 10.1158/2767-9764.CRC-21-0111 (PMC9336209; doi:10.1158/2767-9764.CRC-21-0111)
Supplement: Fig S7 — Fig. S7. Aggregate tSNE plots generated from aggregate analysis of different clusters of WTC and control patients. A, Circled populations in t-SNE plots showed significant differences between WTC and control patients. B, List of markers used to define the clusters identified in tSNE plots (WTC, n=8 and non-WTC, n=12). C, Meta clusters showing the difference in cell density values occurring in IMC tissue samplings (n=2) for each patient sample (n=10). [file crc-21-0111-s07.pdf]

Fig. S7

A

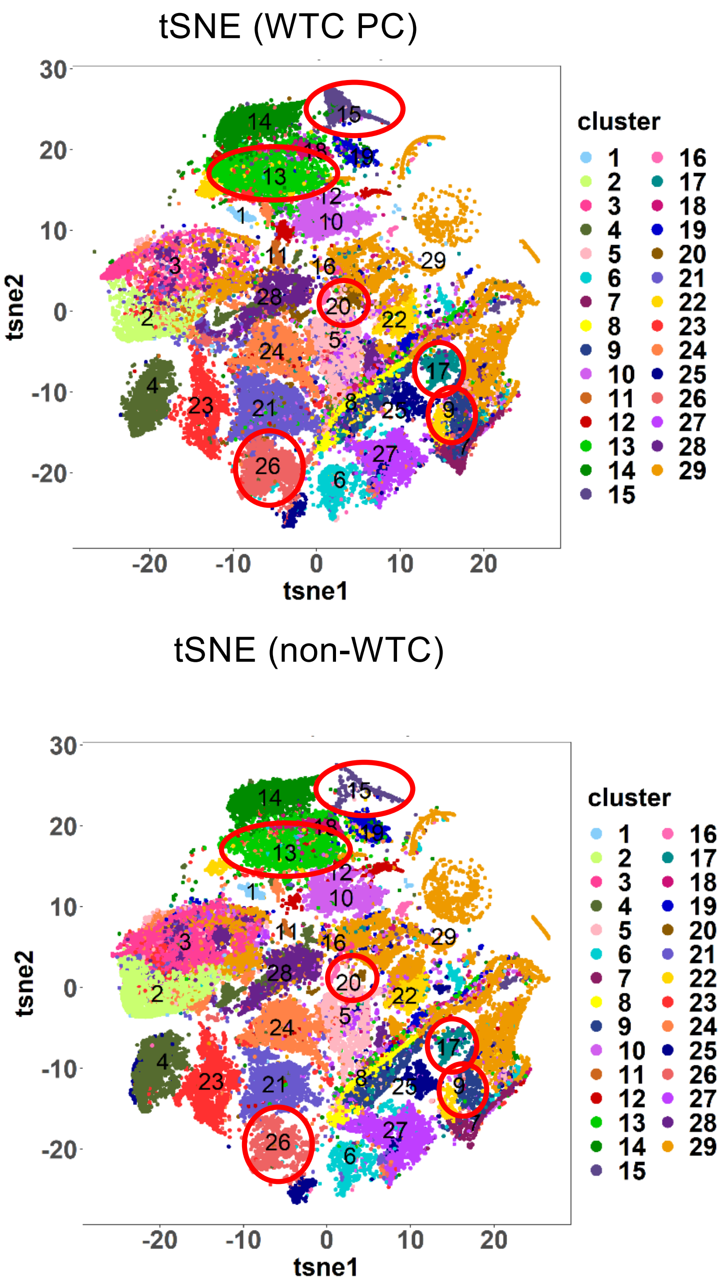

B

Cluster	Cell type
1	Ki67+
2	p504S+PanCK+
3	PanCK+
4	p63+
5	Ecad+
6	CD31+
7	aSMA+
8	Collagen+
9	aSMA+Collagen+
10	CD163+CD206+
11	CD86+
12	CD68+
13	CD4+CD3+CD45+
14	CD8+CD45+CD3+
15	CD20+CD45+
16	CD15+
17	CD56+
18	CD45+
19	CD3lo
20	pp38+
21	HLADR+
22	Foxp3+
23	PD1+
24	pSTAT1+
25	pERK+
26	pAkt+
27	pSTAT3+
28	pNFkB+
29	Surface-

C

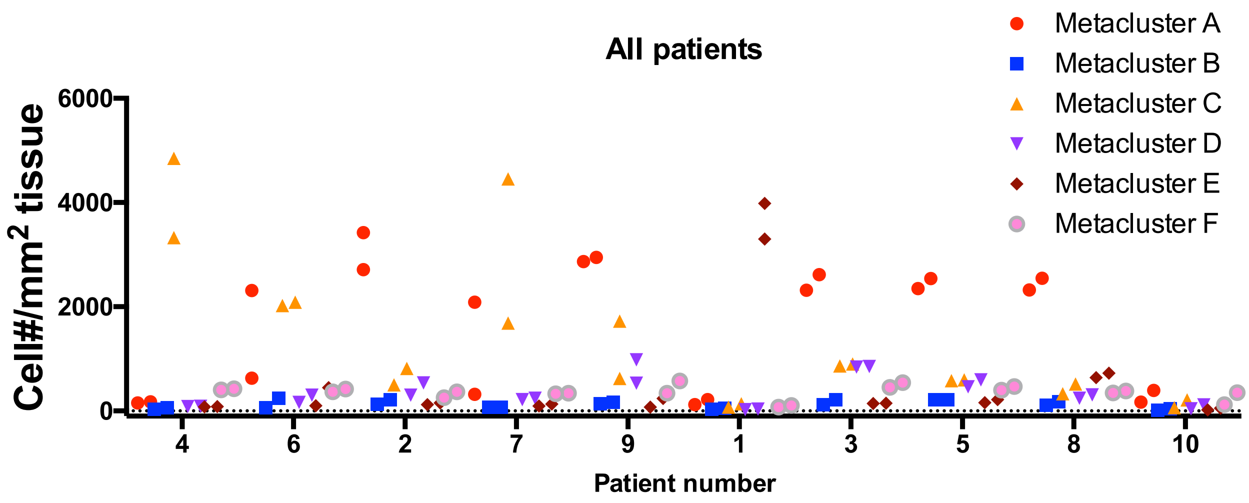

Fig. S7. Aggregate tSNE plots generated from aggregate analysis of different clusters of WTC and control patients. **A**, Circled populations in t-SNE plots showed significant differences between WTC and control patients. **B**, List of markers used to define the clusters identified in tSNE plots (WTC, n=8 and non-WTC, n=12). **C**, Meta clusters showing the difference in cell density values occurring in IMC tissue samplings (n=2) for each patient sample (n=10).
