# Supplementary material for: World Trade Center Dust Exposure Promotes Cancer in PTEN-deficient Mouse Prostates
Source: Cancer Res Commun. 2022 Jun 27;2(6):518–32. doi: 10.1158/2767-9764.CRC-21-0111 (PMC9336209; doi:10.1158/2767-9764.CRC-21-0111)
Supplement: Fig S8 — Fig. S8. Pseudo images showing the spatial distribution between representative clusters in WTC and non-WTC human prostate cancer cohorts. [file crc-21-0111-s08.pdf]

Fig. S8

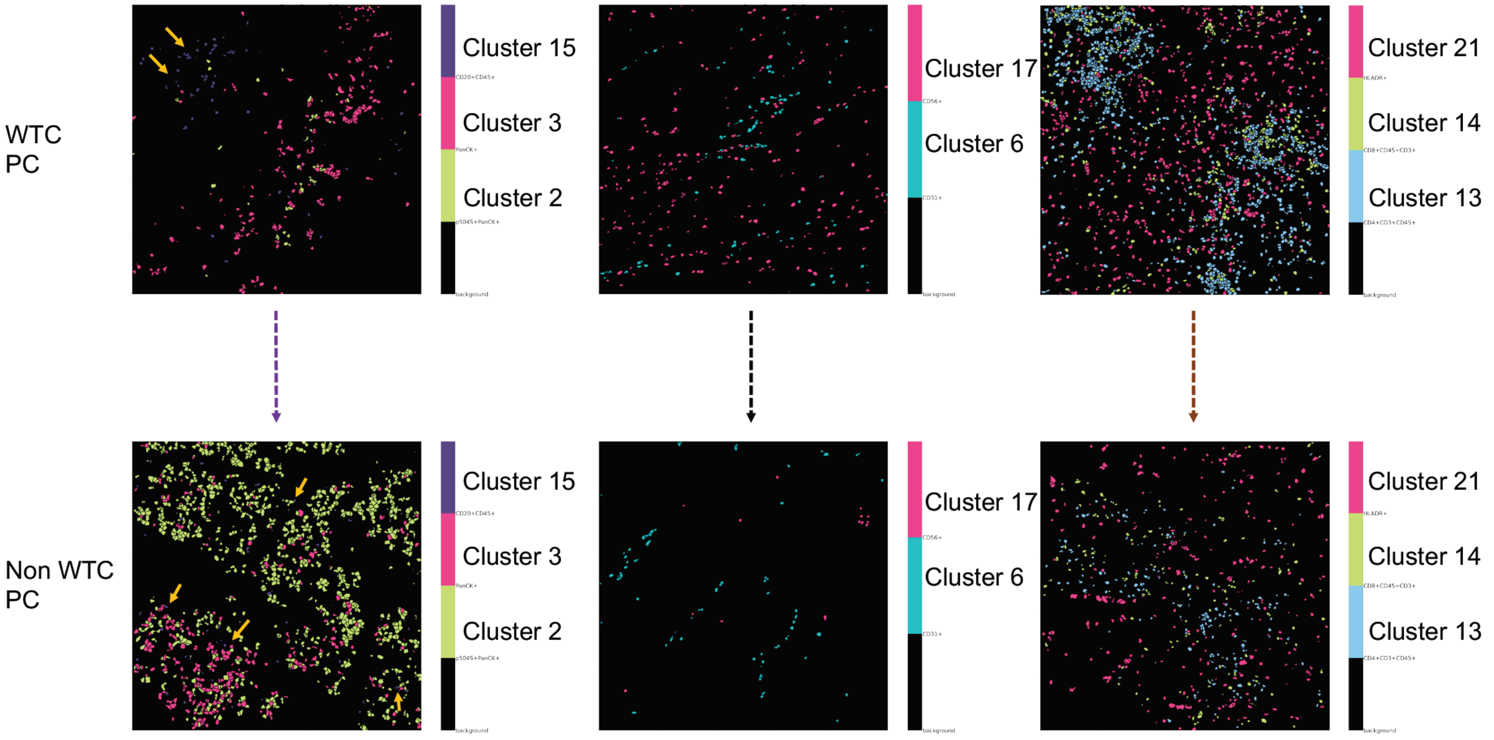
WTC
PC
Non WTC
PC
Cluster 15
Cluster 3
Cluster 2
Cluster 17
Cluster 6
Cluster 21
Cluster 14
Cluster 13
Cluster 15
Cluster 3
Cluster 2
Cluster 17
Cluster 6
Cluster 21
Cluster 14
Cluster 13

Fig. S8. Pseudo images showing the spatial distribution between representative clusters in WTC and non-WTC human prostate cancer cohorts.
